# *Daphnia magna* as an Alternative Model for (Simultaneous) Bioaccumulation and Chronic Toxicity Assessment—Controlled Exposure Study Indicates High Hazard of Heterocyclic PAHs

**DOI:** 10.1021/acs.est.5c00384

**Published:** 2025-04-30

**Authors:** Göksu Çelik, Schylar Alexandra Healy, Stefan Stolte, Philipp Mayer, Marta Markiewicz

**Affiliations:** † Dresden University of Technology, Institute of Water Chemistry, Bergstr. 66, D-01062 Dresden, Germany; ‡ University of Vienna, Centre for Microbiology and Environmental Systems Science, Environmental Geosciences EDGE, 1090 Vienna, Austria; § Technical University of Denmark, Department of Environmental and Resource Engineering, DK-2800 Kongens Lyngby, Denmark

**Keywords:** NSO-PAHs, bioaccumulation, passive dosing, depuration rate constant, chronic toxicity

## Abstract

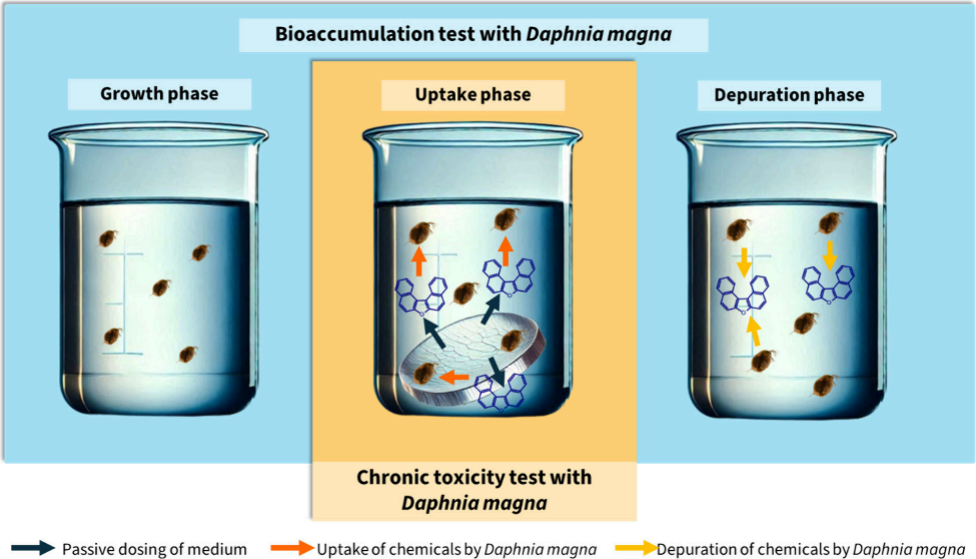

Testing the bioaccumulation and chronic toxicity of (highly) hydrophobic compounds is extremely challenging, but crucial for hazard assessment. Fish are used as a model organism in these tests, but have many limitations, including a long time to reach steady-state, difficulty in maintaining constant exposure, and ethical concerns. We developed a method for the (simultaneous) assessment of chronic reproductive toxicity and bioaccumulation using *Daphnia magna* as a model organism. As test chemicals, we selected heterocyclic polyaromatic hydrocarbons (heterocyclic PAHs), which are often persistent and show high acute aquatic toxicity, raising concerns about their long-term effects. In this study, we developed a robust passive dosing method to maintain constant exposure in chronic toxicity and bioaccumulation tests of four heterocyclic PAHs in *Daphnia magna*. Passive dosing maintained stable exposure concentrations in the ng to μg L^–1^ range, even after reusing disks up to three times. All chemicals were toxic to *Daphnia magna* with EC_10_ values between 0.1 and 15 μg L^–1^. Bioaccumulation tests showed that steady-state was not reached, and the uptake rate constant (*k*_1_) could not be reliably determined due to complex exposure routes (both via water and diet). However, depuration rates in *Daphnia magna* were about 2 orders of magnitude higher than in fish, which is advantageous in the assessment of highly hydrophobic compounds. We propose to use the depuration rate constant (*k*_2_), which is independent of the uptake route, as an indicator of bioaccumulation potential. The *k*_2_ thresholds for *Daphnia magna* were estimated to identify (very) bioaccumulative compounds by correlating *k*_2_ values with bioconcentration factors (BCFs) for *Daphnia magna* and applying fish BCF thresholds. We suggest that a *Daphnia magna* bioaccumulation test can be used as a screening tool to trigger further bioaccumulation testing in fish, as it offers higher throughput, is more ethical, and reaches steady-state faster. However, further validation with reference test protocols and substances is essential.

## Abstract 2

This study proposes a method for (simultaneous) chronic toxicity and bioaccumulation assessment of hydrophobic compounds in *Daphnia magna*, offering a more ethical, efficient alternative to fish tests.

## 1 Introduction

Testing highly hydrophobic chemicals, such as larger heterocyclic polyaromatic hydrocarbons (heterocyclic PAHs > 4 rings in the structure), is not trivial, even in short-term tests due to quick and dramatic decreases in exposure concentration. We have recently shown that in acute tests, significant toxic effects can be observed if exposure is held stable, where none would otherwise occur.^1^ Maintaining constant exposure in chronic toxicity tests is even more challenging because exposure times are longer, organisms are larger and must be fed. Chronic tests often provide only a single value, e.g., 10% effect concentration (EC_10_), no observed effect concentration (NOEC) or lowest observed effect concentration (LOEC)^2^ making the information obtained disproportionately small compared to the effort.

Another shortcoming of toxicity and bioaccumulation testing for hydrophobic chemicals is the long time required to reach equilibrium concentrations in exposed organisms. Based on model by Mackay et al., we calculated that 7 and 344 days would be required for the least and most hydrophobic heterocyclic PAHs investigated in this study to reach 50% of steady-state in a small fish (3 g), while these half-lives would reduce to approximately 3 and 154 days in a smaller fish (0.3 g) (assuming no metabolic transformation in either case).^3^ Given their much smaller size (∼3 mg at 10 days, ∼6 mg at 20 days, and up to ∼8 mg fully grown), water fleas are expected to reach equilibrium even faster.^4^

Lastly, a linear relationship between chemical’s hydrophobicity (log *K*_OW_) and bioconcentration factors (BCFs) has been reported for compounds with log *K*_OW_ of 1–7, which then levels off for superhydrophobic chemicals.^5^ This leveling off is attributed to experimental artifacts caused by the use of total water concentrations instead of freely dissolved concentrations (*C*_free_) in calculation of steady-state BCF (BCF_ss_).^6^ Since hydrophobic chemicals bind to dissolved and particulate organic matter,^7^ using total water concentrations (e.g., measured via liquid–liquid extraction) significantly underestimates BCF.^6^ These effects can be counteracted by the use of passive dosing, as the polymer donor maintains *C*_free_ at stable levels given that the amount of organic matter is small compared to the mass of the polymer.

We selected nitrogen, sulfur, or oxygen-substituted heterocyclic PAHs (NSO-PAHs) as test compounds due to their environmental ubiquity,^8,9^ persistence^10,11^ and high toxicity (acute EC_50_ < 1 mg L^–1^) to aquatic organisms. Continuous emissions mainly from coal and oil industries^12^ may lead to increasing environmental concentrations. The lack of mineralization in standard ready biodegradability tests and even in enhanced tests with adapted microbial communities suggests possible persistence.^11^ Although monitoring data are scarce and often limited to contaminated sites, small heterocycles can reach μg L^–1^ levels in surface waters (see Table S1). Chronic toxicity data for aquatic invertebrates exist for only a few two- and three-ring N-PAHs, showing significant reproductive effects (EC_50_ < 0.1 mg L^–1^ in 21 days).^13^ More hydrophobic NSO-PAHs (log *K*_OW_ > 4) such as 13H-dibenzo[a,i]carbazole, thianthrene, dibenzothiophene, benzo[b]naphtho[2,3-*d*]thiophene, dibenzofuran, xanthene and dibenzo[1,4]dioxane show high bioaccumulation in fish.^14^ Benzo[b]naphtho[2,1-*d*]thiophene and 13H-dibenzo[a,i]carbazole accumulate even more in water fleas.^15,16^

Heterocyclic PAHs can have effects comparable to or more pronounced than their homocyclic counterparts.^17^ For example, some nitrogen- and oxygen-containing compounds induce genotoxicity without enzymatic activation, whereas most homocyclic PAHs cannot damage DNA directly.^18,19^ Carbazole, acridine, dibenzofuran, and xanthene act as weak to moderate aryl hydrocarbon receptor (AhR) agonists,^20^ unlike homocyclic PAHs like naphthalene, fluorene, anthracene and phenanthrene, which do not trigger AhR-mediated toxicity.^21^ Although some specific effects have been reported, the mechanisms of NSO-PAHs toxicity remain largely unknown. Limited aquatic toxicity data hinder comparisons between homo- and heterocycles, particularly as research on the latter is lagging behind. Data on long-term toxicity or bioaccumulation are even more scarce, complicating ecological risk assessment. Despite growing concerns about their environmental impact,^1,11,22,23^ heterocyclic PAHs are not routinely monitored in water and are not included in the priority pollutant lists.^24^

This work aims to determine the chronic toxicity and bioaccumulation of four NSO-heterocyclic PAHs as representative hydrophobic compounds. We hypothesized that

(i) passive dosing provides stable long-term exposure even at very low levels (ng L^–1^) and passive dosing donor can be reused without reloading;
(ii) acute toxicity tests underestimate the hazard posed by highly hydrophobic NSO-PAHs due to slow uptake of these compounds;
(iii) the freshwater invertebrate *Daphnia magna* can be used for bioaccumulation assessments, instead of, or in addition to, fish, offering faster uptake and depuration kinetics and sufficient biomass for reliable chemical quantification.

To test these hypotheses, we designed and conducted separate chronic toxicity and bioaccumulation tests with *D. magna* using passive dosing. We also investigated the potential of combining these tests by using the chronic toxicity test as the uptake phase of the bioaccumulation test, followed by a depuration phase.

## 2 Materials and Methods

### 2.1 Chemicals

The heterocyclic PAHs used in this study, benzo[b]naphtho[1,2-*d*]thiophene (BNT, purity 97%, CAS# 205–43–6), benzo[b]naphtho[1,2-*d*]furan (BNF, >98%, CAS# 205–39–0), and dinaphtho[2,1-b:1′,2′-d]furan (DNF, 97%, CAS# 194–63–8), were purchased from TLC Pharmaceutical Standards (Zwijndrecht, Belgium). 7H-benzo[c]carbazole (BCRB, 97%, CAS# 205–25–4) was acquired from BLD Pharmatech GmbH (Kaiserslautern, Germany). These four chemicals were selected as representative NSO-PAHs due to their occurrence in contaminated aquatic environments, persistence, high hydrophobicity (log *K*_OW_ > 5), and structural diversity (incorporating N, S, or O heteroatoms). Detailed information on the test chemicals is provided in Table 1. Other materials used in this study are listed in the Supporting Information (SI) file, section S1.

**Table 1:**
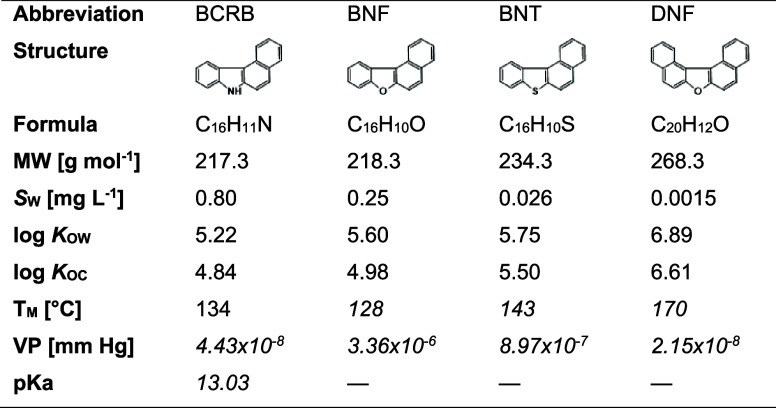
Molecular and Physicochemical Characteristics of Heterocyclic PAHs under Study^a^

a Abbreviations: MW—molecular weight, *S*_W_—water solubility, *K*_OW_—n-octanol–water partition coefficient, *K*_OC_—organic carbon–water partition coefficient, T_M_—melting point, VP—vapor pressure, p*K*_a_—acid dissociation constant. Measured data for log *K*_OW_, log *K*_OC_ and *S*_W_ obtained from our previous work.^22^ Measured data for T_M_ and VP were taken from the EPA Comptox Chemicals Dashboard database^25^ where available; otherwise, predictions (*italics*) were made using the EPISuite MPBPVP model *v1.43*, with the Modified Grain method used for VP.^26^ p*K*_a_ was predicted by COSMO-RS.

### 2.2 Maintenance of *Daphnia magna* Culture

*D. magna* (clone 5, kindly provided by BioChem Agrar GmbH; originally from RWTH Aachen) was grown in beakers containing animals of the same age at a density of 10 animals per liter of M4 medium prepared according to OECD guideline 211.^2^ Animals were fed *Desmodesmus subspicatus* (0.2 mg C/*Daphnia*/day) three times a week. Additional supplements, including spirulina and fish food extract (see SI–S2 for recipe), were fed once a week. The culture was maintained in a climate chamber at a constant temperature (20 ± 1 °C) and light/dark cycle (16:8 h; light intensity of 1 klux).

### 2.3 Passive Dosing System

#### 2.3.1 Casting and Loading

Polydimethylsiloxane (PDMS) passive dosing disks, each weighing 2 g with a surface area of 19.6 cm^2^, were prepared using SYLGARD 184 silicone elastomer kit and cleaned before use. Details of the procedure are given in Section S3 (SI file). PDMS disks were loaded in methanolic solution or suspension of the test compounds (10 mL solution/disk) with constant shaking for 72 h, wiped with a lint-free tissue, and washed with water for 30 min four times to remove methanol. Table S2 lists the concentrations of loading solution in each test.

#### 2.3.2 Dosing

The time required to reach equilibrium between the PDMS disk and the aqueous medium was determined by conducting 72-h kinetic experiments. Chemicals were dosed into M4 medium in 250 mL Schott bottles (in triplicates) at a ratio of 1 disk/100 mL M4 medium using disks loaded with methanolic solution of a single test substance. For the controls, the disks were loaded with pure methanol. While shaking the bottles at room temperature, five samples were taken at different time points over 72 h and the equilibration time was determined.

### 2.4 Chronic Toxicity for *Daphnia magna*

Chronic toxicity tests with *D. magna* were performed according to the OECD guideline 211^2^ using the passive dosing method. Two days before the tests, the exposure solution was predosed by shaking 10 PDMS disks loaded with the test substance in 1 L of medium for 48 h. Each test vessel (250 mL) was first flushed with the dosed solution to equilibrate the vessel walls, then filled with 100 mL of test solution, one PDMS disk, and one juvenile *D. magna* (<24 h old). The tests were conducted in loosely closed bottles to allow for adequate gas exchange and limit evaporation. The exposure medium was changed three times per week, and the animals were fed *D. subspicatus* daily (0.2 mg C/*Daphnia*/day). The number of offspring per adult and behavioral or physiological effects (e.g., size, movement, unhatched eggs) were recorded daily (except weekends). Dissolved oxygen, pH and temperature were also measured each week.

Tests were performed under static conditions (20 °C, 16:8 h light/dark cycle, 1 klux). Each compound was tested in 10 replicates for controls and limit tests (a single high concentration exposure to screen for significant toxic effects) and with 6 or 10 replicates for concentration–response tests. Reuse of silicone disks was included in some experiments (Table S3). Reproductive inhibition was assessed by counting offspring over 21 days. The number of offspring per mother in treatment groups was compared to controls, and the percent inhibition per replicate was used to generate concentration–response curves. A minimum of five concentrations per compound were tested to determine EC_10_, EC_20_, and EC_50_ values. For each test, three sets of polymers were rotated between loading, dosing, and exposure stages (Figure S2). Initial (before animal and food addition) and final (after 2–3 days of exposure, just before the medium change) water concentrations (*C*_W_) were monitored, with the latter collected after centrifugation to remove biomass (e.g., algae, carapaces, or feces).

### 2.5 Bioaccumulation in *Daphnia magna*

Bioaccumulation tests with *D. magna* were adapted from OECD guideline 305 (for fish).^27^ Each compound was tested using 110 animals: 10 for controls and 100 for chemical exposure. The animals, aged 10 days to limit growth dilution and provide sufficient tissue for analysis, were exposed to a single subtoxic concentration of each chemical. The test included three phases: (i) growth, (ii) uptake, and (iii) depuration. A combined chronic toxicity and bioaccumulation test was also conducted in a minimized version (section 2.6).

#### 2.5.1 Growth Phase

Ten days before exposure, 110 juvenile daphnids (<24 h old) were collected from the stock culture and grown in M4 medium. Due to the large number of animals required for this test, the volume of media and amount of food provided to the *Daphnia* were reduced for experimental feasibility. This was achieved by maintaining a denser population (20 mL medium/daphnid), as well as less food (*D. subspicatus* equivalent to 0.1 mg C/daphnid/day) compared to the stock culture yet still within the limits allowed by OECD 211. Other dietary supplements (fish food and spirulina) and physical conditions (temperature, light/dark cycle) were the same as the stock culture.

#### 2.5.2 Uptake Phase

Before exposure started, 2 L of M4 medium containing *D. subspicatus* at a density sufficient for 100 daphnids for 5 days (25 mg C L^–1^) was dosed with the test substance by shaking with 24 PDMS disks for 72 h. For controls (10 animals), 200 mL of M4 medium containing *D. subspicatus* (2.5 mg C L^–1^) was equilibrated with 2 disks loaded with pure methanol. Immediately before the start of exposure phase, 10-day old daphnids were transferred to fresh medium for 1 h to clean their guts. Thereafter, 10 control daphnids were placed in 200 mL of pre-equilibrated clean medium with 2 clean PDMS disks, while 100 animals (treatment group) were divided into two beakers, each with 50 animals, 1 L of predosed exposure medium, and 12 loaded disks to begin the 5-day uptake phase.

Neonates were discarded daily, and animals were sampled at 24 h intervals over 120 h (5 time points, 2 replicates of 5 animals each). Sampled daphnids were transferred to clean medium for 15 min to remove chemicals present on animal surface, dried with lint-free tissue and weighed to determine wet weight. Finally, 5 animals of known mass were combined in each vial and stored at −20 °C until extraction. As passive dosing maintains constant concentrations,^1^ medium samples for analysis (with and without biomass) were only taken at the end of the uptake phase.

#### 2.5.3 Depuration Phase

50 animals were first transferred to fresh medium for 15 min to remove exposure solution from their surface, and then placed in a new clean medium for the depuration phase. Animals were sampled at 4 time points (2 replicates of 5 animals each as in the uptake phase) over 69 to 96 h. After sampling, the remaining animals were transferred to fresh medium, and the old medium was collected for analysis. Control animals and their medium were analyzed at the end of both the uptake and depuration phases (5 animals each).

### 2.6 Simultaneous Bioaccumulation and Chronic Toxicity Test with *Daphnia magna*

To maximize data output, we combined chronic toxicity and bioaccumulation tests in an integrated approach where the chronic toxicity test also served as an uptake phase followed by a depuration/recovery phase (Table S3). This simultaneous bioaccumulation/chronic toxicity test was performed for BNT and DNF. Of the 10 replicates per exposure level, five animals were sampled on day 21 to measure accumulated chemical concentrations and wet weights. The remaining five were transferred to clean media without test chemicals for a 12-day depuration phase under conditions identical with the uptake phase except for the absence of test chemicals. Juvenile counts were recorded daily. At the end of the depuration period, five *Daphnia* were collected, weighed, and analyzed for body burden (i.e., concentration of test chemical per unit of body weight) using the extraction procedure described in Section 2.7.

### 2.7 Chemical Analysis

Heterocyclic PAHs were quantified by liquid–liquid extraction followed by gas chromatography (GC system 7890A) with a mass-selective detector (MS 5975C, Agilent, Waldbronn, Germany) (see SI file section S4 for details). For chronic toxicity tests, aqueous samples from replicates were pooled when necessary (i.e., for BNT and DNF at exposure concentrations below 0.3 μg L^–1^) to meet LOQ. The surrogate standards were spiked into the (centrifuged) aqueous samples before extraction with hexane. The hexane extracts were dried with Na_2_SO_4_, transferred to GC vials, and the internal standard (pyrene in hexane, 50 μg L^–1^ in the final sample) was added. The list of surrogate and internal standards, along with the limit of detection (LOD: 2–3 μg L^–1^) and the limit of quantification (LOQ: 6.1–8.9 μg L^–1^) for each test substance, is provided in Table S4.

For quantification of body burdens, 5 daphnids were thawed, homogenized in 5 mL Milli-Q water (IKA T-25 digital Ultra-TURRAX) and extracted twice with 2 mL of hexane. The extracts were combined, dried with Na_2_SO_4_, concentrated under a nitrogen stream, and spiked with internal standard.

### 2.8 Data Analysis

All data are presented as average ± standard deviation. Student’s *t* test was used to analyze significance (*p*-value >0.05 considered insignificant). Concentration–response curve parameters (effective concentrations and confidence limits) and plots were generated using GraphPad Prism software (V6.01, GraphPad Software, La Jolla, CA, USA). The exposure concentrations were log_10_ transformed before fitting. Then a four parameters logistic model with variable slope was used to obtain EC_10_, EC_20_ and EC_50_ (see section S5 in SI file for details). Effective chemical activities (EA_50_) for chronic toxicity were calculated as follows:
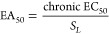
1

EA_50_ is the effective activity responsible for 50% of the effect, and EC_50_ (effect concentration at which 50% effect is observed compared to control) and *S*_L_ (subcooled liquid solubility) are expressed in nmol L^–1^. Subcooled solubility *S*_L_ was calculated according to eq 2.^28^
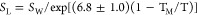
2

Here, *S*_W_ is water solubility (nmol L^–1^), T is the system temperature (293.15 K) and T_M_ is the chemical’s melting point ([K]).

The concentration of the test chemical in the lipid of *D. magna* at which 10% inhibition of reproduction is observed, known as the critical target lipid body burden (CTLBB [μmol g^–1^ octanol]), was calculated using the updated target lipid model for chronic end points^29^ with a universal slope m= −0.94, chronic EC_10_ [mmol L^–1^], and a correction factor for PAHs Δc = 0.352 (eq 3)
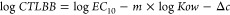
3

The depuration rate constant (*k*_2_) was derived by plotting the natural logarithm of *C*_D_ against time ([h]), with the slope giving *k*_2_ (Figure S3).^27^ The growth rate constant (*k*_g_) was determined from the slope of the regression line in the plot of ln (*D. magna* wet weight) versus time, and the growth-corrected depuration rate constant (*k*_2g_) was calculated using eq 4.
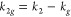
4

From *k*_2_, the optimal uptake and depuration durations to reach 50% and 95% steady-state were estimated using eqs 5 and 6.
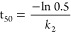
5
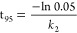
6

The bioaccumulation factor (BAF*) was calculated, even though steady-state was not reached. This BAF is used for comparison and should not be considered equivalent to the steady-state BAF.
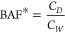
7*C*_D_ in eq 7 represents the concentration in *D. magna* at the end of the uptake phase (μg kg^–1^ wet weight), while *C*_W_ is the concentration in water (μg L^–1^). To account for lipid content differences among organisms, BAF* values were normalized to a standard 5% lipid content (characteristic for fish^27^) using eq 8,^27^ yielding the lipid-normalized BAF* (BAF*_L5%_).
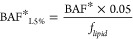
8where *f*_lipid_ is the actual lipid fraction in the organism (assumed to be 1.5% for *D. magna*(30)), and “0.05” represents 5% lipid content.

## 3 Results and Discussion

### 3.1 Maintaining Constant Exposure in Chronic Toxicity Tests with *Daphnia magna*

**Figure 1:**
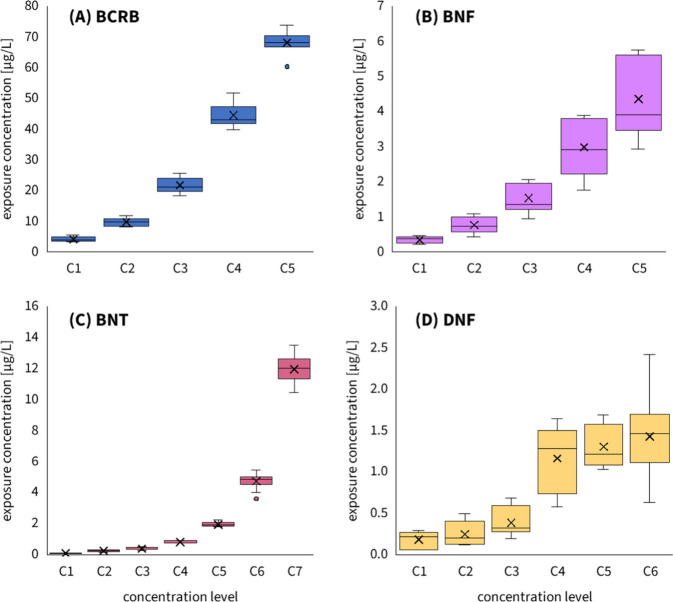
Measured exposure concentrations (*C*_W_ – water concentrations after removal of biomass) during chronic toxicity tests reported as the average of all measured concentrations (both initial and final *C*_W_) during all subsequent media change cycles (*n* = 9–12). Each box represents a different concentration level of the corresponding test substance (BCRB, BNF, BNT, and DNF) and shows the mean (× symbol), median (horizontal middle line in each box), the lower quartile (Q1; lower end of the box), and the upper quartile (Q3; upper end of the box). Whiskers show the minimum and maximum values. Data outside the Q1-Q3 range are shown as outliers (circles).

The equilibrium in passive dosing of medium was achieved within 48 h for all heterocycles (Figure S4) and remained stable even after adding animals and food (Figure 1) across a range of test concentrations varying by 3 orders of magnitude (70 ng L^–1^ for BNT to 68 μg L^–1^ for BCRB). Variability in measured concentrations was less than 7% at the higher end and 21% at the lower end of this range (expressed as coefficient of variation, CV); the measured concentrations of BNT and BCRB are shown in Figure S5 and S6. During a single cycle of medium change, no systematic decrease was observed between the initial (freshly dosed medium) and final (just before medium change) *C*_W_ at or below the solubility limit of the test compounds. Consequently, exposure concentrations were reported as the average of all sampling points collected over the 21-day period. The results confirm that the passive dosing system effectively compensated for concentration losses of heterocycles with log *K*_OW_ ranging from 5.2 to 6.9, which could have otherwise reached up to 100% loss.^1^ Parkerton et al. observed no toxic effects for chemicals with log *K*_OW_ 4.7–7.9 using passive dosing in 21-day chronic limit toxicity tests with *D. magna*.^31^ In our study, this method was extended to concentrations below saturation.

Since reusing polymers for passive dosing reduces labor, costs, and waste generation, we assessed the viability of reusing PDMS disks in chronic toxicity testing. In tests with BCRB and BNF, the same set of polymers achieved similar concentrations after three reuse cycles without reloading (see Figure 1 and Figure S6), eliminating the need for repeated polymer loading.

### 3.2 Chronic Toxicity Test with *Daphnia magna*

All chronic toxicity tests met the validity criteria (parent mortality <10%, average number of live offspring per mother >60 in controls, and CV for the number of live offspring in controls <15%).^2^ The biocompatibility of PDMS disks was confirmed, with no statistically significant difference (*p*-value >0.05) in the number of live offspring produced per adult between control samples containing polymer loaded in pure methanol (68 to 86 offspring in five tests) and quality control tests without polymer (68 and 76 offspring in two tests).

Heterocyclic PAHs inhibited the reproduction of *D. magna* at concentrations as low as 9.8 μg L^–1^ for BCRB, 3.0 μg L^–1^ for BNF, 0.8 μg L^–1^ for BNT, and 0.2 μg L^–1^ for DNF (the lowest observed effect concentrations causing statistically significant reproduction inhibition compared to controls – LOEC, with *p*-value <0.05). Table 2 shows the concentration–response test results, including effective concentrations (EC_10_, EC_20_ and EC_50_). According to REACH Annex XIII, BNF, BNT and DNF are classified as “toxic to aquatic life” (chronic EC_10_ < 10 μg L^–1^), while the least hydrophobic compound, BCRB, had a slightly higher EC_10_ of 15 μg L^–1^. Toxicity (based on EC_10_) increases with the hydrophobicity of the compounds (BCRB < BNF < BNT < DNF). There is an effect of heteroatom type, with the N-containing BCRB being the least toxic of the 4-ring compounds. The S-containing BNT is slightly more toxic than the O-containing BNF. The same trend in toxicity (BCRB < BNF < BNT based on EC_10_) was previously observed in acute tests with green algae and *D. magna*.^1^ Although based on a small number of compounds, a more general structure-activity relationship can be formulated that S-containing compounds are the most toxic and N-containing compounds the least toxic within the homologue series. Such rules can be used as a weight of evidence approach in the evaluation of data-poor compounds.^1^

**Table 2:**
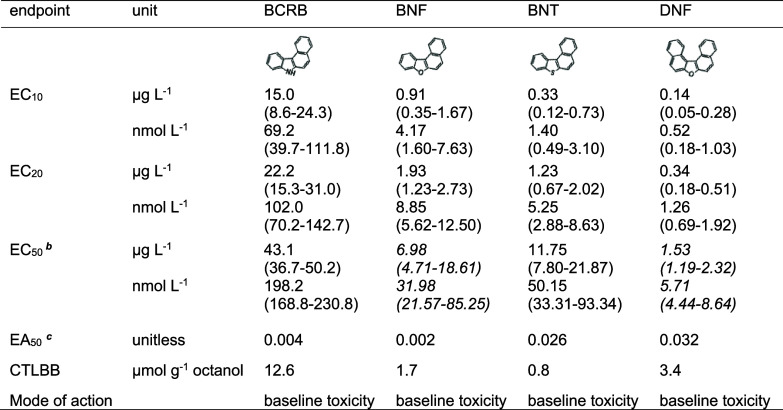
Results of Chronic Reproduction Tests with *D. magna*^a^

a Effective concentrations (EC_*x*_) are reported in μg L^–1^ and nmol L^–1^ with 97.5–2.5% confidence intervals given in brackets. Effective activities (EA_50_) were calculated using eq 1, and CTLBB was derived from EC_10_ using eq 3. The concentration–response curves for the chronic toxicity tests are given in Figure S7.

b EC_50_ values for BNF and DNF were extrapolated from concentration–response curves as they fell outside of the tested concentration range.

c Baseline toxicity generally occurs at activities between 0.001 and 0.01 for chronic end points.^32^

Effective activity (EA_*x*_) and critical target lipid body burden (CTLBB) were used to assess whether the tested compounds might possess specific modes of action in chronic test with *D. magna*. According to literature, EA_50_ values for acute endpoints typically fall within the range of 0.01–0.1 for baseline toxicity^28^ (i.e., toxicity caused by nonspecific intercalation of chemicals into biological membranes, leading to loss of membrane structure and impaired functioning), while values for chronic endpoints are expected to be lower, ranging from 0.001 to 0.01.^32^ Similarly, CTLBB values for chronic toxicity across 36 species range from 0.36 to 137 μmol g^–1^ octanol, with an estimated value of 4.3 ± 1.3 μmol g^–1^ octanol for *D. magna*,^29^ while another study reported values of 0.12 to 7.5 μmol g^–1^ lipid for *Ceriodaphnia dubia* and homocyclic PAHs.^33^ The EA_50_ values for BCRB and BNF determined in this study fall within the range characteristic of baseline toxicity, whereas those for BNT and DNF are slightly higher. Additionally, the CTLBB values for NSO-PAH observed in this study were consistent with that characteristic of baseline toxicity in chronic tests with *D. magna* (see Table 2).

In our previous study, we observed acute effects on *D. magna* immobilization for BCRB, BNF and BNT (for the latter, 35% effect at water solubility), whereas no effects were observed for DNF, even under controlled exposure conditions.^1^ Since the time required to reach 95% steady-state for BNF, BNT, and DNF (Table 3) exceeds the 48-h acute *D. magna* immobilization test duration,^34^ the lack of acute effects is probably due to too short exposure time. As a result, a longer time is required for such hydrophobic organics to reach sufficient body burdens to exert toxicity, and conducting short-term toxicity tests is not always meaningful for such compounds.^35^ Additionally, the lack of acute toxicity for highly hydrophobic chemicals can be explained by their limited solubility in membranes and aqueous media or the inability to reach sufficiently high chemical activity in test media.^28,35^

**Table 3:**
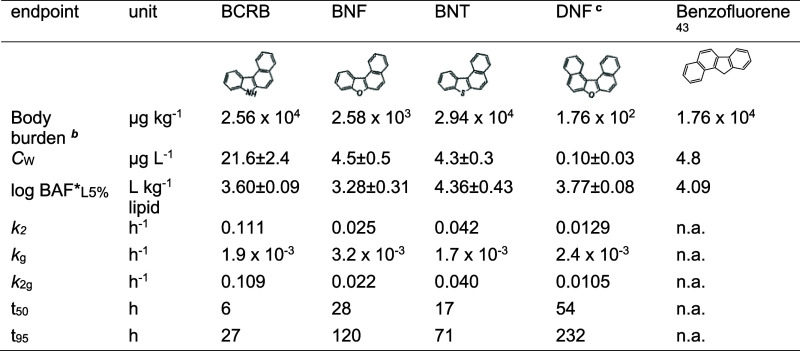
Results of Bioaccumulation Tests with *D. magna*^a^

a Abbreviations: *C*_W_—concentration in water (after centrifugation of algae); BAF*_L5%_—lipid-normalized bioaccumulation factor (calculated as for steady-state) corrected to the lipid content of 5% wet weight and assuming 1.5% is the actual lipid content of daphnids using eq 8; *k*_2_—depuration rate constant; *k*_g_—growth rate constant; *k*_2g_—growth dilution corrected depuration rate constant; t_50_—time to reach 50% steady-state; t_95_—time to reach 95% steady-state; n.a.—not available.

b Body burden refers to the chemical concentration in animal body at the end of the uptake phase (*t* = 120 h).

c Kinetic parameters derived for DNF should be interpreted with caution due to large variations in *C*_D_ and the limited number of available data points during depuration.

We also monitored the following endpoints: presence of unhatched eggs, offspring mortality, body weight of parent organisms, and unusual swimming behavior. Unhatched eggs were negligible (<1%), but at higher exposure levels, 20 to 42% offspring mortality occurred in BCRB, BNT, and DNF treatments (BNF caused <1% offspring mortality, Figure S8). BCRB, BNF, and BNT significantly inhibited *D. magna* growth (*p*-value <0.05), but DNF did not except at saturation (Figure S9). Additionally, the two highest exposure concentrations of BCRB (22 and 68 μg L^–1^) induced locomotor changes, including hyperactivity, continuous spinning, rapid maneuvers, and reduced vertical migration.

To increase the information output of testing, we sought to perform chronic toxicity and bioaccumulation tests in a single effort. During the 21-day exposure, fertility decreased, and BNT and DNF accumulated in daphnids, with body burdens of 6.9 mg kg^–1^ for DNF (*C*_W_ = 1.4 μg L^–1^). For BNT, measured at two different exposure concentrations, body burdens were 50 mg kg^–1^ (*C*_W_ = 4.7 μg L^–1^) and 150 mg kg^–1^ wet weight (*C*_W_ = 12 μg L^–1^). Assuming steady-state conditions, log BAF*_L5%_ was 4.20 for DNF and 4.58 ± 0.05 for BNT. Compared to controls, reproductive inhibition ranged from 36 to 57% during exposure (days 1–21), but decreased to 6–15% during recovery (days 21–33) (Figure S10). At the end of the depuration phase, the body burdens were below the LOQ, which means that *Daphnia* removed at least 99.57% of the accumulated body burden of DNF and 99.98% of BNT after 12 days of depuration. The small size and rapid growth of daphnids at the beginning of chronic toxicity tests requires a very sensitive analytical method to measure tissue concentrations and is confounded by growth dilution, which often cannot be measured reliably. Therefore, we developed a separate method using older daphnids for easier handling (section 3.3). Using a more sensitive analytical device (LOQ ≪ 6–9 μg L^–1^) would allow simultaneous chronic toxicity and bioaccumulation testing without significantly increasing the effort.

### 3.3 Bioaccumulation in *Daphnia magna*

No mortality was observed in the control groups, and no traces of the test chemicals were detected in the animals or medium. No exposure-related parental mortality was observed in the treatment groups either. Even though exposure concentrations were chosen to provide measurable levels of test chemicals in the animals without being lethal, statistically significant effects on growth (*p* < 0.05, based on wet weight) were noted in animals exposed to 21.6 μg L^–1^ BCRB and 4.5 μg L^–1^ BNF compared to controls maintained in beakers containing polymer disks loaded with pure methanol (Figure S11). No inhibitory growth effects were seen in tests with BNT (4.3 μg L^–1^) and DNF (0.1 μg L^–1^). Passive dosing enabled successful quantification of exposure concentrations after centrifugation of algal biomass (*C*_W_), closely matching freely dissolved concentrations (*C*_free_) calculated from partition coefficients between methanol and the medium (*K*_MeOH:medium_; see Figure S12 and section S6 for details).

**Figure 2:**
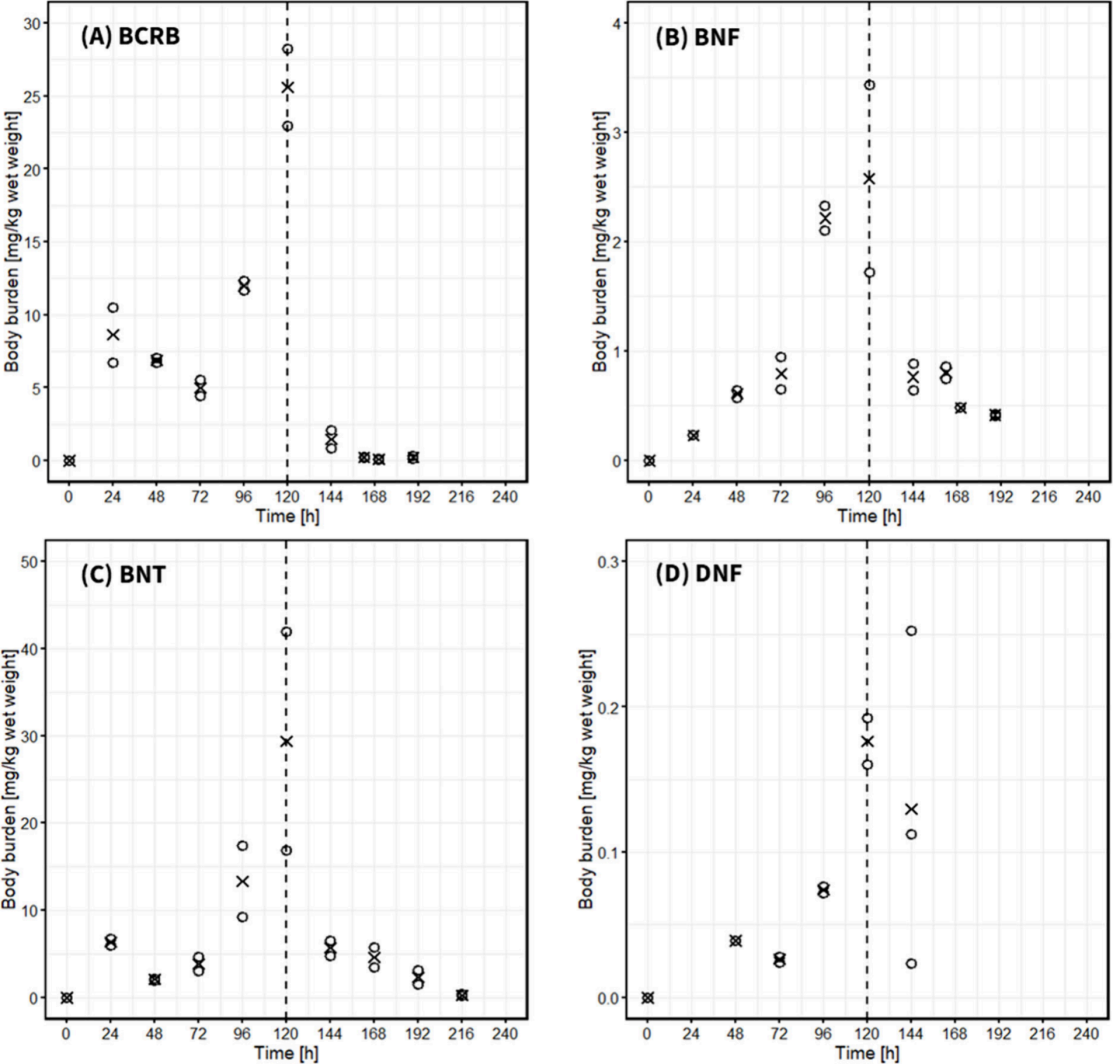
Body burdens of NSO-PAHs in *D. magna* (in mg kg^–1^ wet weight) over time. The uptake phase ended after 120 h and was followed by the depuration phase. Data points marked with “×” represent mean measured concentration in *D. magna*, while open circles indicate individual measurements.

All heterocyclic PAHs bioaccumulated in *D. magna.* The time to reach 95% steady-state (t_95_) for chemical uptake, calculated using equations for one-compartment bioaccumulation model for fish,^27^ increased in the order: BCRB < BNT < BNF < DNF, while the half-life (t_50_) for chemical depuration was under 54 h (Table 3). Similar time frames have been reported for water fleas for compounds of similar hydrophobicity.^15,16,36−42^ Although the durations of the uptake (120 h) and depuration (69 or 96 h) phases were theoretically sufficient to reach equilibrium (except for DNF), we observed an ongoing increase in chemical concentrations in daphnid bodies during the uptake phase (Figure 2). This indicates that, at least for the NSO-heterocycles investigated in this study, the theoretical assumptions valid for fish or water fleas are not satisfied.^4,27^

NSO-PAHs concentrations in *D. magna* rapidly increased during the first 24 h of exposure (Figure 2). After the initial increase, body burdens of BCRB, BNT and, to a lesser extent, DNF decreased from 24 to 72 h before rapidly rising again up to 120 h. This phenomenon has been observed in previous studies on the bioaccumulation of estrone,^42^ pyrene^41^ and perfluoroalkyl acids (PFAs)^44^ in *D. magna* and may be due to (1) offspring delivery, (2) metabolism (difficult to judge for NSO-PAHs due to lack of data, but few studies showed biotransformation of homocyclic PAHs e.g. pyrene in *D. magna*(40,45,46)) or (3) slow kinetics (slow transport of the chemical into internal circulation causing delayed elimination^44^). Body burdens of the heterocycles continued to increase during the exposure phase, and none reached steady-state within the 5-day uptake phase, indicating that the assumption of steady-state within 24 h may underestimate the bioaccumulation potential of hydrophobic chemicals in *D. magna*. The lipid-normalized bioaccumulation factor (BAF*_L5%_) given in Table 3 should therefore not be considered as equivalent to the steady-state BAF, but rather as a best-case scenario approximation of bioaccumulation potential (meaning that the actual BAF is probably higher). This is especially true for BNF and DNF for which the calculated BAF*_L5%_ are lower than expected based on the log *K*_OW_ of the compounds or depuration rates (see Figure 4). Furthermore, in simultaneous chronic toxicity and bioaccumulation tests, *Daphnia* showed higher accumulation of BNT and DNF after 21 days of exposure, with log BAF*_L5%_ values of 4.58 and 4.20, respectively. These values exceed those obtained from the stand-alone bioaccumulation tests, which involved only 5 days of exposure and resulted in log BAF*_L5%_ values of 4.36 for BNT and 3.77 for DNF. This suggests that for highly hydrophobic substances such as DNF, longer exposure durations may be required to approach steady-state.

In the absence of steady-state, the kinetic bioaccumulation and biomagnification factors (BCF_k_ and BMF_k_) should be calculated. Arnot and Quinn reported that steady-state was either not confirmed or not reached in 60% of the fish bioaccumulation data they collected (n = 869).^47^ For highly hydrophobic chemicals, the presence of even small amounts of organic matter in medium can have a large impact on the concentration in water and lead to an underestimation of the BCF_ss_, necessitating the use of rather complex analytical procedures (e.g., passive sampling or solid phase microextraction) to measure *C*_free_. If the exposure concentration is kept constant during the uptake phase (e.g., by passive dosing), the calculation of BCF_k_ does not require actual measured aqueous concentrations (as these are constant and equal to the initial concentration), which increases its relevance for hydrophobic chemicals, but requires more frequent sampling than needed to obtain BCF_ss_. Existing protocols for bioaccumulation assessment consider either gill or dietary uptake, making uptake by both routes difficult to handle.^48^ Calculation of uptake kinetics is also not always straightforward (see Figure S13 for details), even in the case of bioconcentration via water (can be quite error-prone if it is slow),^49^ and is even more difficult in dietary studies, where factors like uptake routes, feeding behavior, or food preferences can affect uptake rates.^50^

Since *D. magna* must feed continuously to maintain healthy reproduction rates, and since accumulation of the test compound in algae is unavoidable with the use of passive doing method,^51^ we exposed *D. magna* to chemicals via both contaminated food and water, i.e., we measured bioaccumulation (and not only bioconcentration). This is in contrast to standard fish bioaccumulation tests, where feeding is discrete, and removal of food shortly after feeding (30 min to 1 h) to limit uptake of the chemical to almost exclusively aqueous exposure is possible.^27^

Unlike uptake rate, depuration rate is independent of exposure route and is therefore considered to be a more reliable metric for assessing bioaccumulation potential than BCF and BMF values.^50,52^ Goss et al. suggest to omit estimating the uptake rate and to use elimination half-lives (t_50_) derived from depuration rate constants (*k*_2_) as a metric to assess bioaccumulation.^50^ The growth-corrected depuration rates (*k*_2g_) for *D. magna* ranged from 0.011 to 0.109 h^–1^ and increased in the order DNF < BNF < BNT < BCRB. Due to relatively large variations in *C*_D_ and the limited number of available data points for DNF during depuration, these data should be treated with caution (Figure 2). Furthermore, the 192 h data point for BCRB was excluded from the depuration model fit due to the apparent plateau in elimination. This suggests possible presence of elimination-resistant residues, which may arise from slow elimination processes within specific compartments, such as internal storage or binding to specific biomolecules.^53^ This could extend retention beyond the primary elimination phase. At the end of the depuration phase, body burdens of BCRB, BNF and BNT in daphnids ranged from 230 to 416 μg kg^–1^ (wet weight).

Invertebrates theoretically require less time to reach steady-state than fish,^4^ and their use in hazard assessment is more ethical and resource-efficient. Although any environmental species can, in principle, be used for bioaccumulation assessment in regulatory context, fish bioaccumulation remains the gold standard. Therefore, comparing bioaccumulation data between *D. magna* and fish species is valuable for evaluating the suitability of water fleas for bioaccumulation assessment.^54^ Schlechtriem et al. suggested using epibenthic amphipod *Hyalella azteca* for bioaccumulation assessment which was recently accepted as an alternative testing method for regulatory purposes by ECHA.^55,56^

**Figure 3:**
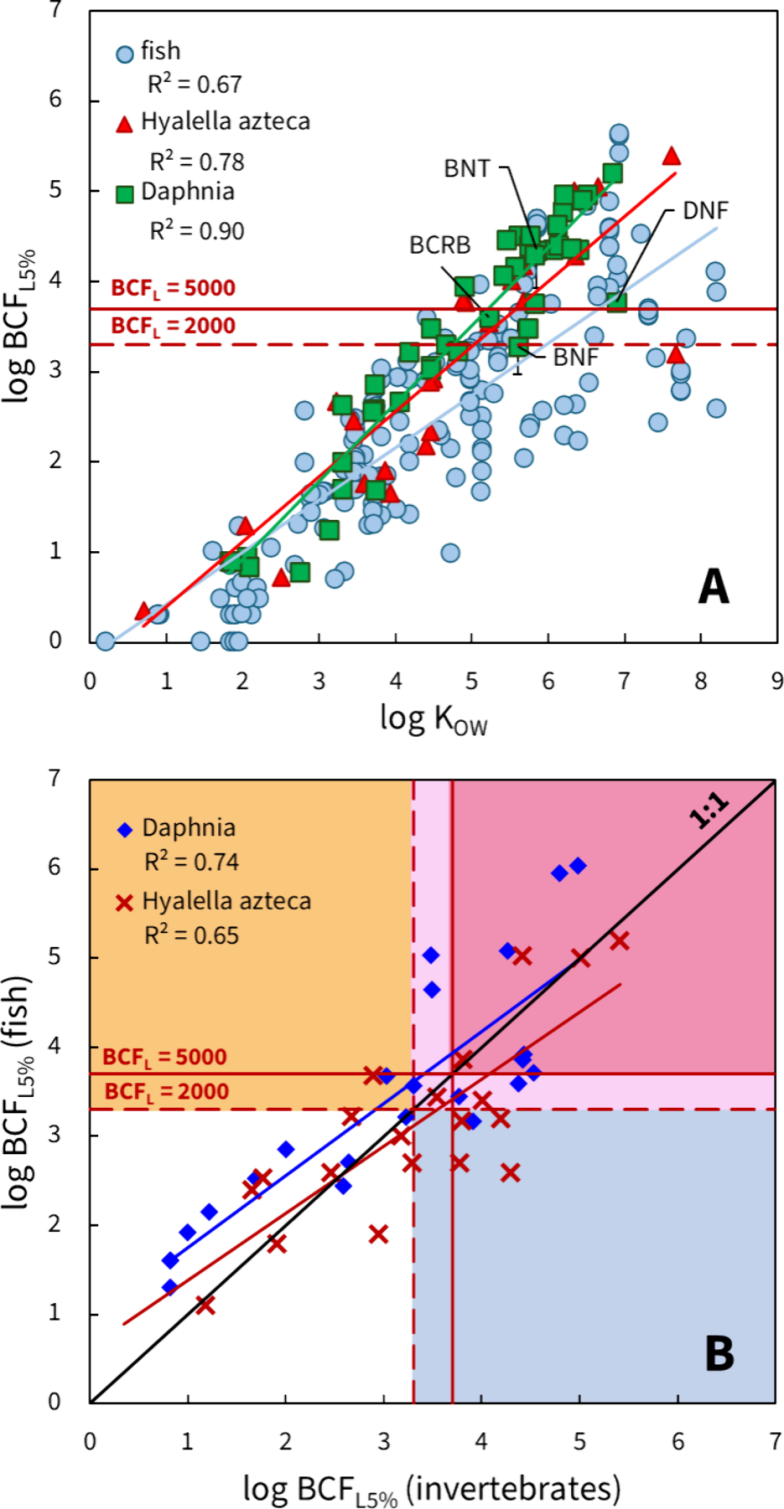
Lipid-normalized log BCF values (BCF_L5%_) in aquatic species. **A** Correlation between log *K*_OW_ and log BCF_L5%_ in fish (*y* = 0.58*x* – 0.15; *R*^2^ = 0.67, *n* = 169), *H. azteca* (*y* = 0.72*x* – 0.33; *R*^2^ = 0.78, *n* = 25) and *Daphnia sp*. (*y* = 0.86*x* – 0.80; *R*^2^ = 0.90, *n* = 45). **B** Correlation between log BCF_L5%_ in fish versus *Daphnia sp*. (*y* = 0.81*x* + 0.94; *R*^2^ = 0.74, *n* = 23) and *H. azteca* (*y* = 0.76*x* + 0.62; *R*^2^ = 0.65, *n* = 20), where upper right area covers chemicals that accumulate in both fish and invertebrates (light and dark pink areas for B and vB categories), the upper left area (orange) indicates chemicals that accumulate in fish but not in invertebrates, and the lower right area (blue) indicates chemicals that accumulate in invertebrates but not in fish.

To compare bioaccumulation in these organisms—*D. magna* or *D. pulex*, *H. azteca* and fish—we compiled BCF data from the literature.^4,7,57−66,14−16,41,43,52,54,55^ Due to the limited availability of data, particularly for invertebrates, both steady-state and kinetic BCF_L5%_ data were used. To facilitate interspecies comparisons, actual lipid content (e.g., 1.5% for *D. magna*, as defined in eq 8) was considered before normalizing all values to 5% lipid. However, once a standardized bioaccumulation assessment for *D. magna* is established, a biologically realistic lipid content of e.g. 1.5% should be used as the default value to avoid uncertainty in BCF values due to variable lipid content. The log *K*_OW_ of compounds correlated better with the log BCF_L5%_ in *Daphnia* (*R*^2^ = 0.90) than in fish (*R*^2^ = 0.67) or *H. azteca* (*R*^2^ = 0.78, Figure 3A). The scatter in the experimental data and poor correlation of BCF with parameters describing hydrophobicity (particularly evident in fish) may be caused by metabolic transformation or other factors causing instability (not reflected by hydrophobicity), difficulty in maintaining and measuring exposure concentrations, or unjustified assumption of steady-state.

A good correlation was found between the log BCF_L5%_ values measured in *Daphnia sp.* and fish (*R*^2^ = 0.74), similar to that between *H. azteca* and fish (*R*^2^ = 0.65, Figure 3B). Nineteen substances were not bioaccumulative in both fish and invertebrates (white area in Figure 3B). Out of 19 data points showing high accumulation in fish (log BCF_L5%_ ≥ 3.3), 17 also accumulate in invertebrates (light and dark pink areas), while the two exceptional data points both correspond to phenanthrene, which accumulated in fish but not in *Daphnia* and *H. azteca*. Five points (four homocyclic PAHs and methoxychlor) in the lower right area (blue) show substances accumulating in invertebrates but not in fish. This could be the result of different metabolic capacities, i.e., PAHs being biotransformed by fish^51,67^ to higher extent than by daphnids.^51,68^ Benzo[a]anthracene (log *K*_OW_ = 5.8) accumulates more in crustaceans (log BCF_L5%_ = 4.5 in *Daphnia*),^4^ than in fish (log BCF_L5%_ = 3.7)^54^ as is the case for many other PAHs.^54^ For this reason, bioaccumulation in *Daphnia* could serve as a conservative screening approach (“worst-case scenario”). Our analysis shows that bioaccumulation classifications in fish and *D. magna* are the same for 91% of the compounds, while this agreement is 75% between fish and *H. azteca*.

The UK Environment Agency, using BCF data for fish, derived depuration rate constant (*k*_2_) thresholds to classify chemicals as bioaccumulative (B, BCF_L5%_ ≥ 2000 L kg^–1^) or very bioaccumulative (vB, BCF_L5%_ ≥ 5000 L kg^–1^) using REACH criteria. These thresholds correspond to *k*_2_ ≤ 0.0059 h^–1^ (elimination half-life, t_50_ = 117 h or 4.9 days) for B, and *k*_2_ ≤ 0.0027 h^–1^ (t_50_ = 253 h or 10.5 days) for vB, with all BCF values normalized to 5% lipid content.^52^ However, depuration rates in *D. magna* are much higher than those in fish, meaning that none of the tested NSO-PAHs would meet B or vB classification based on fish-derived *k*_*2*_ thresholds, even though BAF*_L5%_ clearly indicates bioaccumulation. This highlights that use of nonfish species (already allowed under REACH) requires different, organism-specific B/vB thresholds to avoid potential underestimation of bioaccumulation risks.

**Figure 4:**
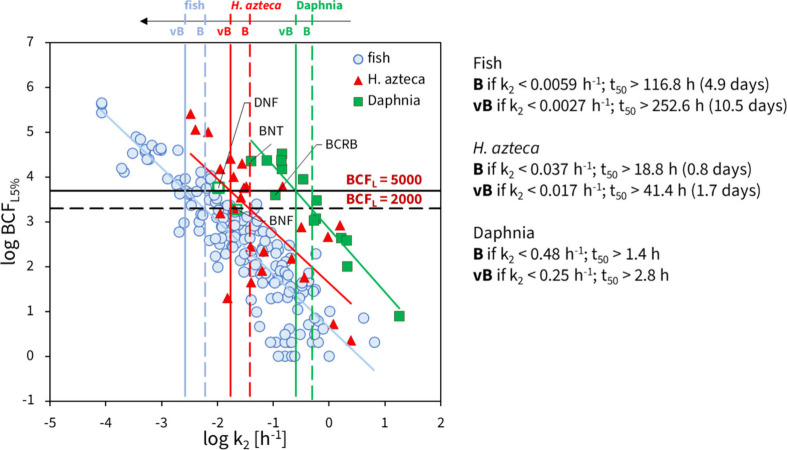
Lipid normalized log BCF_L5%_ values of organic contaminants against the logarithm of the depuration rate constants (log *k*_2_) for *Daphnia sp.* (*y* = −1.41*x* + 2.85; *R*^2^ = 0.90, *n* = 14), *Hyalella azteca* (*y* = −1.16*x* + 1.64; *R*^2^ = 0.49, *n* = 25), and fish (*y* = −1.19*x* + 0.66; *R*^2^ = 0.78, *n* = 169). The bioacccumulative (B) and very bioaccumulative (vB) criteria correspond to BCF_L5%_ values of 2000 and 5000 L kg^–1^, respectively. Vertical lines represent the log *k*_2_ thresholds corresponding to the B (dashed) and vB (solid) criteria for fish (blue), *H. azteca* (red) and *Daphnia sp*. (green). BNF and DNF are shown but were excluded from the correlation due to the fact that the BCF*_L5%_ is probably considerably underestimated (t_95_ equal to or higher than the duration of the uptake phase).

The relationship between the log BCF_L5%_ values of organic chemicals and their depuration rate constants (log *k*_2_) in *Daphnia sp.*, *H. azteca* and fish (Figure 4) show that depuration rates are higher in daphnids, followed by amphipods and then fish, likely due to smaller organisms having higher ventilation rates and surface-to-volume ratios. From Figure 4, we derived *k*_2_ thresholds for *Daphnia sp*. and *H. azteca* corresponding to BCF_L5%_s of 2000 (B) and 5000 (vB). In *Daphnia sp.*, the *k*_2_ values for B and vB criteria are 0.48 h^–1^ (t_50_ = 1.4 h) and 0.25 h^–1^ (t_50_ = 2.8 h), classifying all tested heterocyclic PAHs as vB. For *H. azteca*, the B and vB criteria correspond to lower *k*_2_ values of 0.037 h^–1^ (t_50_ = 18.8 h) and 0.017 h^–1^ (t_50_ = 41.4 h), respectively. Although *k*_2_ values in *H. azteca* tend to be higher than in fish, for superhydrophobic chemicals, reaching steady-state in *H. azteca* may also take more than one month, exceeding standard test durations.^69^ In contrast, *k*_2_ values in *Daphnia sp*. are approximately 20–25 times shorter, making testing more feasible. Due to the small number of data points for both invertebrates, these *k*_2_ thresholds should be treated as a first approximation. A larger, more detailed data set is needed to consider them for regulatory use.

### 3.4 Environmental Significance and Improvement of Testing Methods

Predicted No Effect Concentrations (PNECs), defined as the concentration below which no adverse effects on organisms are expected, were estimated for heterocyclic PAHs using chronic toxicity EC_10_ values and an assessment factor (AF) of 100.^70^ The resulting PNECs ranged from 5 to 692 pmol L^–1^ (1 to 150 ng L^–1^) (see Table S11). Risk Quotients (RQs), calculated as the ratio of environmental concentrations (ranging from 0.8 to 600 μg L^–1^) to PNECs based on our previous approach,^1^ indicated that for the four tested NSO-heterocycles, RQs were significantly greater than 1, suggesting potential risks to aquatic life. However, more data on environmental concentrations of NSO-PAHs are needed for more accurate risk assessments, as the data used by us are rather representative of contaminated sites.

To ensure a precautionary approach, an AF of 100 was applied, which accounts for interspecies variability and uncertainties in extrapolating laboratory findings to real-world conditions. A lower AF of 10 is sometimes used for baseline toxicants, but even then RQ values would still exceed 1, reinforcing the concern that these heterocyclic PAHs pose ecological risks.

All four NSO-PAHs tested in this study were highly bioaccumulative and chronically toxic to *D. magna*, despite showing no or limited effects in prior acute tests.^1^ This suggests that toxicity assessments based solely on acute tests for highly hydrophobic compounds (log *K*_OW_ ≥ 5.9) may underestimate their hazard. Given their high persistence,^11^ these substances should be monitored in waters and biota. The passive dosing method was expanded to achieve stable exposures below water solubility even in complex long-term laboratory experiments reducing the need for polymer reloading and frequent sampling for exposure monitoring.

This study showed that the freshwater crustacean *D. magna* can be used as a model organism for bioaccumulation testing. European environmental regulations focus on bioaccumulation in fish, but recently accepted the use of *Hyalella azteca* (HYBIT test) as an alternative bioaccumulation assessment method.^55,56,62,71^ This work shows that even smaller organisms can and should be used instead of, or in addition to, fish to improve our understanding of chemical bioaccumulation and perhaps support environmental regulation. The use of daphnids has the added advantages that (i) these animals are already well-established in toxicity testing and regulatory practice, (ii) with proper experimental design and sensitive analytical methods, both chronic toxicity and bioaccumulation potential can be assessed in a single experiment, (iii) only 10 days of growth is sufficient before bioaccumulation tests to reduce growth dilution, and (iv) given the faster depuration rates, the organisms can reach equilibrium more rapidly than *H. azteca* or fish. Despite promising results, the use of invertebrates, especially *D. magna*, in bioaccumulation assessment requires larger data sets including different chemical classes for broader validation before regulatory acceptance can be considered.

## Supporting Information Available

Detailed methods (compound information, analytical techniques, experimental procedures, data analysis), supplementary data (environmental concentrations, chronic toxicity test results, GC/MS method details, data sets for Figures 3 and 4), and additional figures (photographs of experimental setups, bioaccumulation kinetics, offspring count) (PDF)
Outcomes of chronic toxicity tests (XLSX)
