# Supplementary material for: Daphnia magna as an Alternative Model for (Simultaneous) Bioaccumulation and Chronic Toxicity Assessment—Controlled Exposure Study Indicates High Hazard of Heterocyclic PAHs
Source: Environ Sci Technol. 2025 Apr 30;59(18):8984–96. doi: 10.1021/acs.est.5c00384 (PMC12080252; doi:10.1021/acs.est.5c00384)
Supplement: Supplementary file 1 — es5c00384_si_001.pdf [file es5c00384_si_001.pdf]

# Supporting Information (SI)

# *Daphnia magna* as an Alternative Model for (Simultaneous) Bioaccumulation and Chronic Toxicity Assessment – Controlled Exposure Study Indicates High Hazard of Heterocyclic PAHs

Göksu Çelik,[1,2] Schylar Alexandra Healy,[1] Stefan Stolte,[1] Philipp Mayer[3] and Marta Markiewicz[1]

*[1] Dresden University of Technology, Institute of Water Chemistry, Bergstr. 66, D-01062 Dresden, Germany.*

*[2] University of Vienna, Centre for Microbiology and Environmental Systems Science, Environmental Geosciences EDGE, 1090 Vienna, Austria*

*[3] Technical University of Denmark, Department of Environmental Engineering, DK-2800 Kongens Lyngby, Denmark*

contains 27 pages, seven text sections (S1-S7), thirteen figures (Figure S1-S13), and eleven tables (Table S1-S11)

**Table 1** Sources, predicted no effect concentrations (PNEC) and environmental concentrations of NSO-PAHs.

| substance | sources | lowest PNEC* [µg $L^{-1}$] | environmental concentrations |
|---|---|---|---|
| | | **N-PAHs** | |
| **indole** 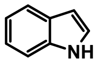 | tobacco smoke, coal tar, perfumery, flower oils, synthetic flavor, cleaning and household products, air freshener, including candles with a fragrance | 2.08 | **26 mg $L^{-1}$** in untreated coke plant wastewater<br>**22 µg $g^{-1}$** in creosote-contaminated sediments<br>**<10–600 µg $L^{-1}$** in surface water<br>**<20-87 µg $kg^{-1}$** in plant (Japanese honeysuckle)<br>**1.72-2.28 µg $L^{-1}$** in water boiled duck meat and duck fat<br>**0.18 ng $m^{-3}$** in air |
| **carbazole** 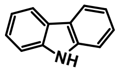 | UV-sensitive photographic plates, dyes, insecticides, lubricants, explosives, rubber antioxidants, odor inhibitor in detergents, coal tar | 0.26 | **270-290 µg $kg^{-1}$** in soil<br>**0.007-24 mg $L^{-1}$** in groundwaters near contamination sites<br>**0.5-5.3 ng $L^{-1}$** in drinking water<br>**0.02-19.4 µg $L^{-1}$** in surface water<br>**4-14.1 ng $g^{-1}$** in soil<br>**0.04-1700 mg $kg^{-1}$** in sediment<br>**0.8-50 ng $m^{-3}$** in air<br>**0.2-3.5 mg $g^{-1}$** in plant near chemical factory<br>**110 µg $L^{-1}$** in fish (bullhead catfish)<br>**3 g $kg^{-1}$** in creosote |
| **benzo[c]carbazole** 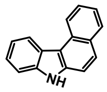 | crude oils, heavy petroleum products | 0.017 | n.a. |
| **benzo[a]acridine** 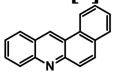 | coal tar, coke oven emissions, cigarette smoke and condensate, synthetic coal fuels, creosote mixtures (BACR isomers) | 0.016 | n.a. |
| | | **S-PAHs** | |
| **dibenzothiophene** 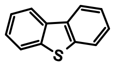 | asphalt tar, coal, tires, cosmetic, pharmaceuticals, acne drug products | 0.24 | **11.2-12.6 mg $kg^{-1}$** in contaminated soils<br>**10 µg $L^{-1}$** in groundwater<br>**9.6-292 µg $kg^{-1}$** in mussel<br>**2.3-12.3 ng $L^{-1}$** in surface waters<br>**0.4-2.3 ng $L^{-1}$** in rainwater<br>**0.03-1300 ng $g^{-1}$** in sediment<br>**0.03-0.46 ng $g^{-1}$** in margarine, butter and vegetable oils<br>**0.002-14 ng $m^{-3}$** in air |
| **thioxanthene** 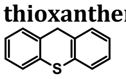 | cosmetics and antipsychotic drugs (TXT derivatives) | 0.32 | n.a. |
| **benzo[b]naphtho[1,2-d]thiophene** 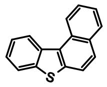 | tar, pitch, tires, rubber products, bitumen, furnace black | n.a. | **380.2 mg $kg^{-1}$** in coal-tar pitch<br>**126.6 mg $kg^{-1}$** in spare tire<br>**1.3 mg $kg^{-1}$** in flip-flop<br>**0.04 mg $kg^{-1}$** in rubber boots<br>**0.03 mg $kg^{-1}$** in bitumen |

**Table 1** continues.

| substance | sources | lowest PNEC* [µg L$^{-1}$] | environmental concentrations |
|---|---|---|---|
| **O-PAHs** | | | |
| dibenzofuran 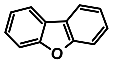 | coal tar, creosote, wood preservatives, heat-transfer oils, dyes, printing textiles, antioxidant in plastics, tobacco smoke, coal gasification | 0.47 | **10-76 ng m$^{-3}$** in air<br>**5.7 mg kg$^{-1}$** in soil near wood processing plant<br>**0.94-41.3 µg kg$^{-1}$** in sediment<br>**0.8-424 µg L$^{-1}$** in groundwaters near creosote contaminated sites<br>**9.9–26 ng L$^{-1}$** rainwater<br>**1-41 ng L$^{-1}$** in surface waters<br>**0.43-56.2 ng L$^{-1}$** in drinking waters |
| **benzo[b]naphtho[1,2-d]furan** 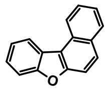 | tar, pitch, carbon black, tires, flip-flop, rubber boots, furnace black, bitumen | n.a. | **474.9 mg kg$^{-1}$** in coal-tar pitch<br>**73.4 mg kg$^{-1}$** in spare tire<br>**0.89 mg kg$^{-1}$** in flip-flop sandals<br>**0.08 mg kg$^{-1}$** in rubber boots |
| **dinaphtho[2,1-b:1',2'-d]furan** 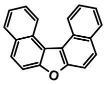 | n.a. | n.a. | n.a. |

n.a. – not available. *The PNEC values were obtained from NORMAN database. Data for sources taken either from NORMAN Database, Hazardous Substances Data Bank (HSDB), Drugbank Online, EPA CompTox Chemicals Dashboard, or References .[1,2] Environmental concentrations were taken from Hazardous Substances Data Bank or Reference [2].

**S1. Chemicals and Materials.** Pyrene (99%, Dr. Ehrenstorfer) was used as the internal standard, while benzo[a]acridine (99.8%, European Commission Joint Research Centre), fluoranthene (98%, Sigma-Aldrich) and benzo[k]fluroanthene (99%, in acetone, Restek) were used as surrogate standards in extraction. Organic solvents, hexane (97%) and methanol (≥99.9%), were purchased from VWR Chemicals.

**S2. Preparation of food supplements for *Daphnia magna* culture:** Spirulina powder was obtained from Steinberger GmbH, Germany, while fish food (Tetra Goldfish Crisps) was purchased from Tetra GmbH, Germany. To prepare dietary supplements for Daphnia culture, specified ratios of spirulina powder (2 g per liter of water) and fish food (5 g per liter of water) were added to water. The fish food suspension was sonicated, allowed to settle, and the resulting supernatant was used to feed the daphnids. To each beaker containing 5 Daphnia, 1 mL of spirulina and 1 mL of supernatant from fish food were added weekly.

**S3. PDMS Passive dosing polymer casting**

Casting began by mixing ten parts of a silicone elastomer base with one part of a catalyst using the SYLGARD™ 184 Silicone Elastomer Kit (Polydimethylsiloxane (PDMS)). The polymer was then poured into aluminum weighing dishes (5 cm in diameter) in 2 ± 0.05 g portions, ensuring an even distribution over the entire surface. After oven drying at 110°C for 30 min, the cured polymers were refluxed in hexane, methanol, and water for 8 h each time, with each solvent being changed three times during the washing process. Subsequently, the disks were dried at 70 °C for 4 hours. Throughout the washing procedure, approximately 5% of the initial mass was lost, and the average mass of a ready-to-use disk was determined as 1.90 ± 0.05 g. The casting and washing processes are shown in Figure S1.

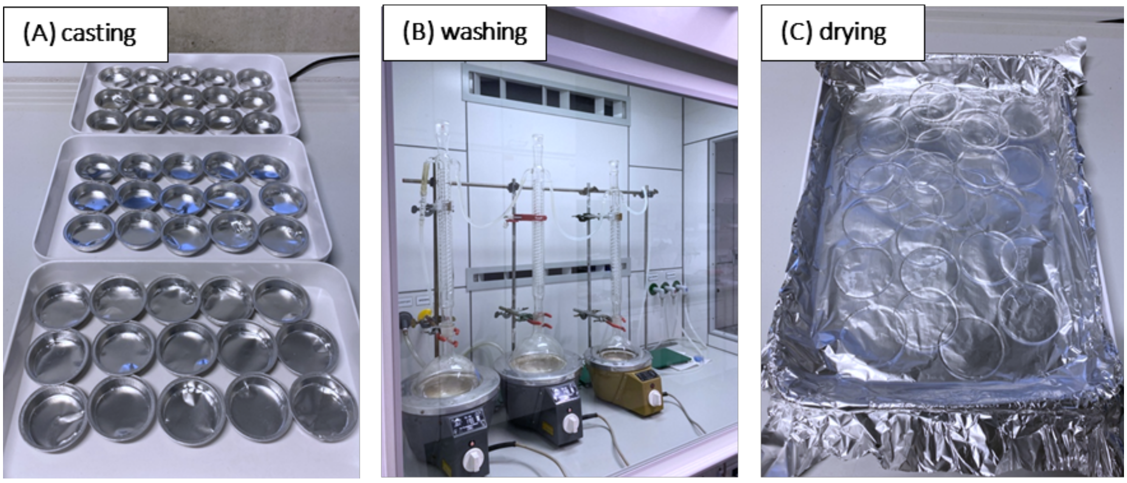


**Figure S1** Preparation of PDMS disks in three steps: (**A**) casting in aluminum weighing dishes, (**B**) washing with hexane, methanol and water sequentially under reflux, and (**C**) oven baking to dry.

**Table S2** Loading solution concentrations of PDMS disks used to dose chemicals in aqueous medium for *D. magna* chronic toxicity tests

| **Chemicals** | **Concentration of methanolic loading solutions [g L$^{-1}$]** | | | | | | |
|---|---|---|---|---|---|---|---|
| BCRB | 0.063 | 0.125 | 0.25 | 0.5 | 1 | | |
| BNF | 0.063 | 0.125 | 0.25 | 0.5 | 1 | | |
| BNT | 0.031 | 0.063 | 0.125 | 0.25 | 0.5 | 1 | 2.5 |
| DNF | 0.125 | 0.25 | 0.5 | 1 | 1.5 | saturated | |

**Table S3** Description of the chronic toxicity experiments conducted in this study, including a test at saturation (limit test) and tests to obtain concentration-response curves (CRC).

| **nr.** | **experiment** | **test compound** | **concentration levels tested** | **replicates in treatment/control group** | **reuse of PDMS disks without reloading** [b] | **depuration/ recovery period** |
|---|---|---|---|---|---|---|
| 1-a [a] | limit test | DNF | 1 | 10/10 | no | yes |
| 1-b [a] | CRC | BNT | 2 | 10/10 | no | yes |
| 2 | CRC | DNF | 5 | 6/10 | no | no |
| 3 | CRC | BNT | 5 | 6/10 | no | no |
| 4 | CRC | BNF | 5 | 6/10 | x3 times | no |
| 5 | CRC | BCRB | 5 | 6/10 | x3 times | no |

[a] Tested in the same experimental run. [b] Polymer reuse refers to the repeated use of loaded polymers (3 times) without reloading in methanolic solutions to achieve the same exposure concentration during subsequent media changes during chronic tests.

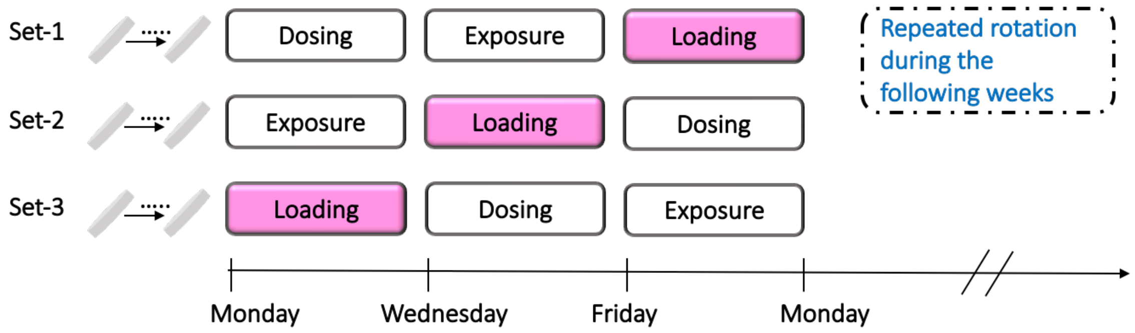


**Figure S2** Rotation of the three sets of polymers during the 3-week chronic toxicity tests following stages: (i) loading from methanolic solutions, (ii) dosing the medium, and (iii) exposure to the test organisms. In the tests with BNF and BCRB, each set of polymers was reused consecutively three times (without reloading) during the three-week exposure period.

## S4. Details of instrumental analysis

For GC/MS analysis, samples (1 µL) were injected using an autosampler in pressure-pulsed splitless mode. A Restek Rxi-5Sil MS capillary column (5% diphenyl/95% dimethylpolysiloxane, 30 m x 0.25 mm; 0.25 µm film thickness) was used with helium as the carrier gas at a flow rate of 1.6 mL min$^{-1}$. The GC method parameters were: inlet temperature at 315 °C, oven program at 80 °C (held for 1.6 min), then ramp to 320 °C at 50 °C min$^{-1}$. The MS operated at 70 eV ionization energy, with the source at 250 °C and quadrupole at 180 °C. The results were analyzed using Chemstation software (Agilent Technologies, Germany). Concentrations were calculated based on peak areas normalized by the corresponding internal standard and a five-point calibration series. Table S4 provides GC/MS method characterization details, including LOD and LOQ.

**Table S4** Characterization of the GC/MS method for the quantification of heterocyclic PAHs [a]

| # rings | test substance | LOQ/LOD [µg L$^{-1}$] | mass-to-charge ratio (m/z) | surrogate standard | internal standard |
|---|---|---|---|---|---|
| 4-rings | BCRB | 7.3/2.4 | **217**, 216, 189 | benzo[a]acridine | pyrene |
| | BNF | 6.1/2.0 | **218**, 189, 219 | fluoranthene | |
| | BNT | 8.6/2.9 | **234**, 235 | | |
| 5-rings | DNF | 8.9/3.0 | **268**, 239, 269 | benzo[k]fluoranthene | |

[a] m/z given in bold represents quantifier ion, while the other ions are qualifier ions.

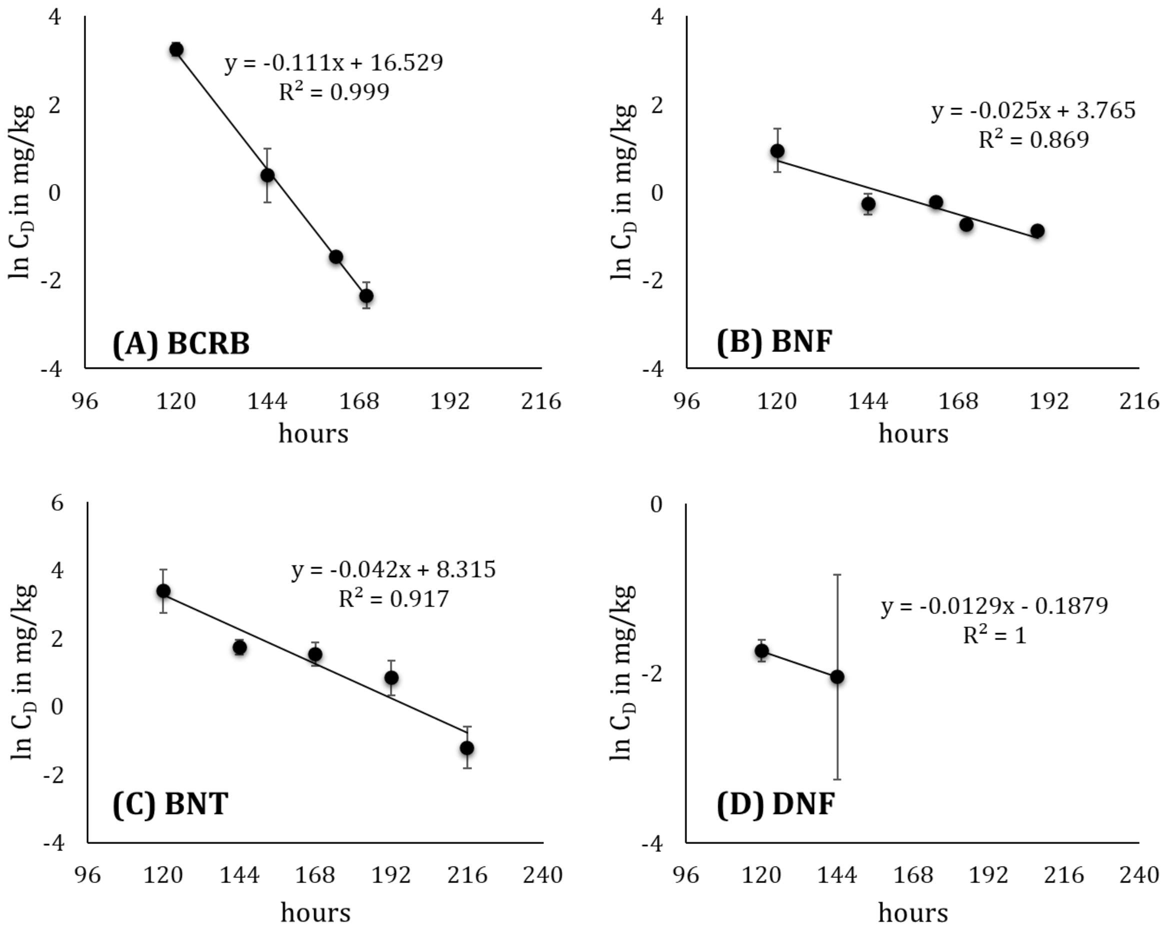


**Figure S3** Depuration rate constants of heterocyclic PAHs in bioaccumulation tests with *D. magna*, determined from the slopes of the curves drawn as the natural logarithm of chemical concentration in *Daphnia* against time. Due to instrumental errors in the analysis of later samples, only the first two time points during the depuration phase were available for DNF, resulting in a higher uncertainty. For BCRB, the 192 h time point was excluded from the model fit as an outlier due to the presence of elimination-resistant residues in *D. magna*.

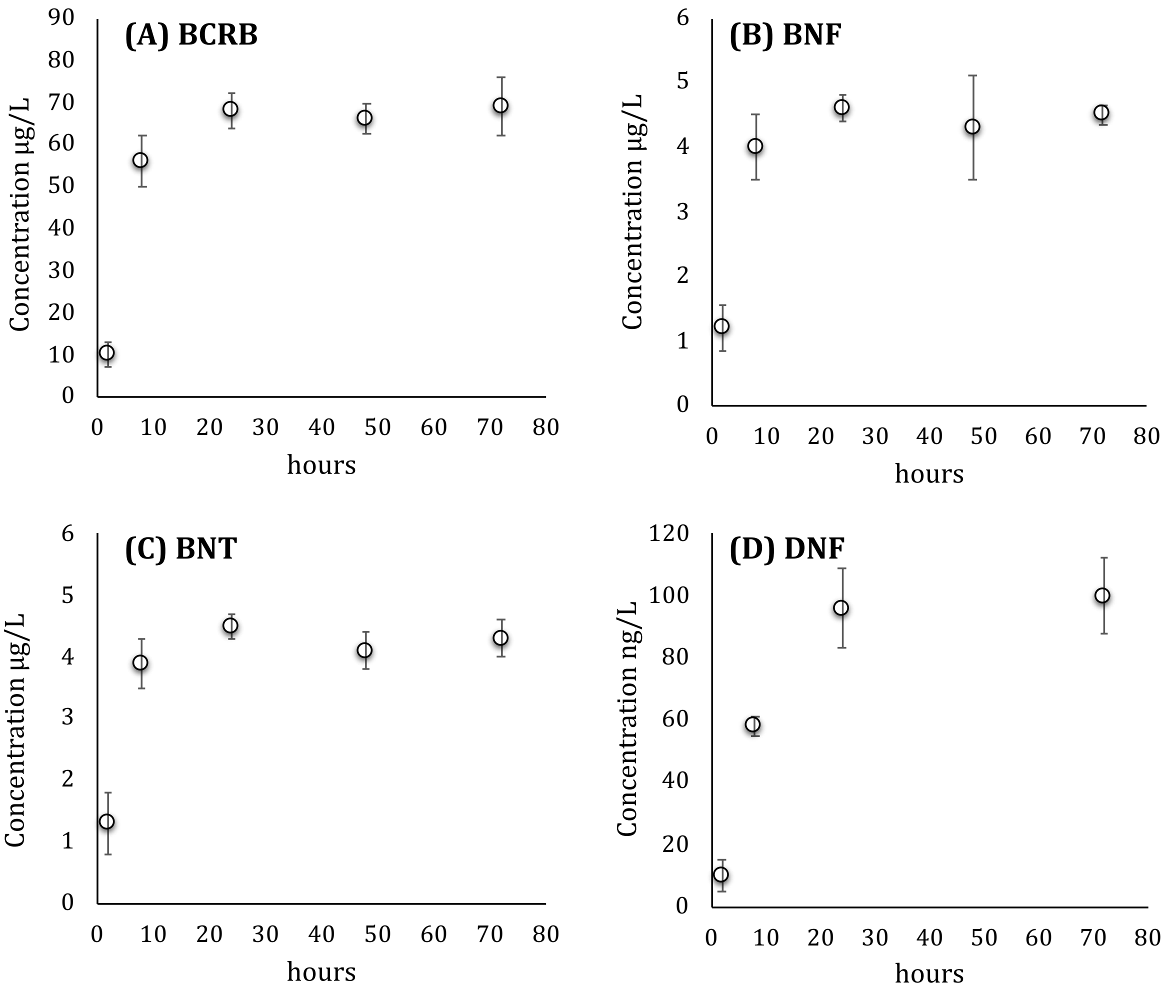


**Figure S4** Release kinetics of four heterocyclic PAHs from one loaded PDMS disk into 100 mL aqueous medium. Loading solution concentrations were 1 g/L for BCRB, BNF and BNT, and 100 mg/L for DNF.

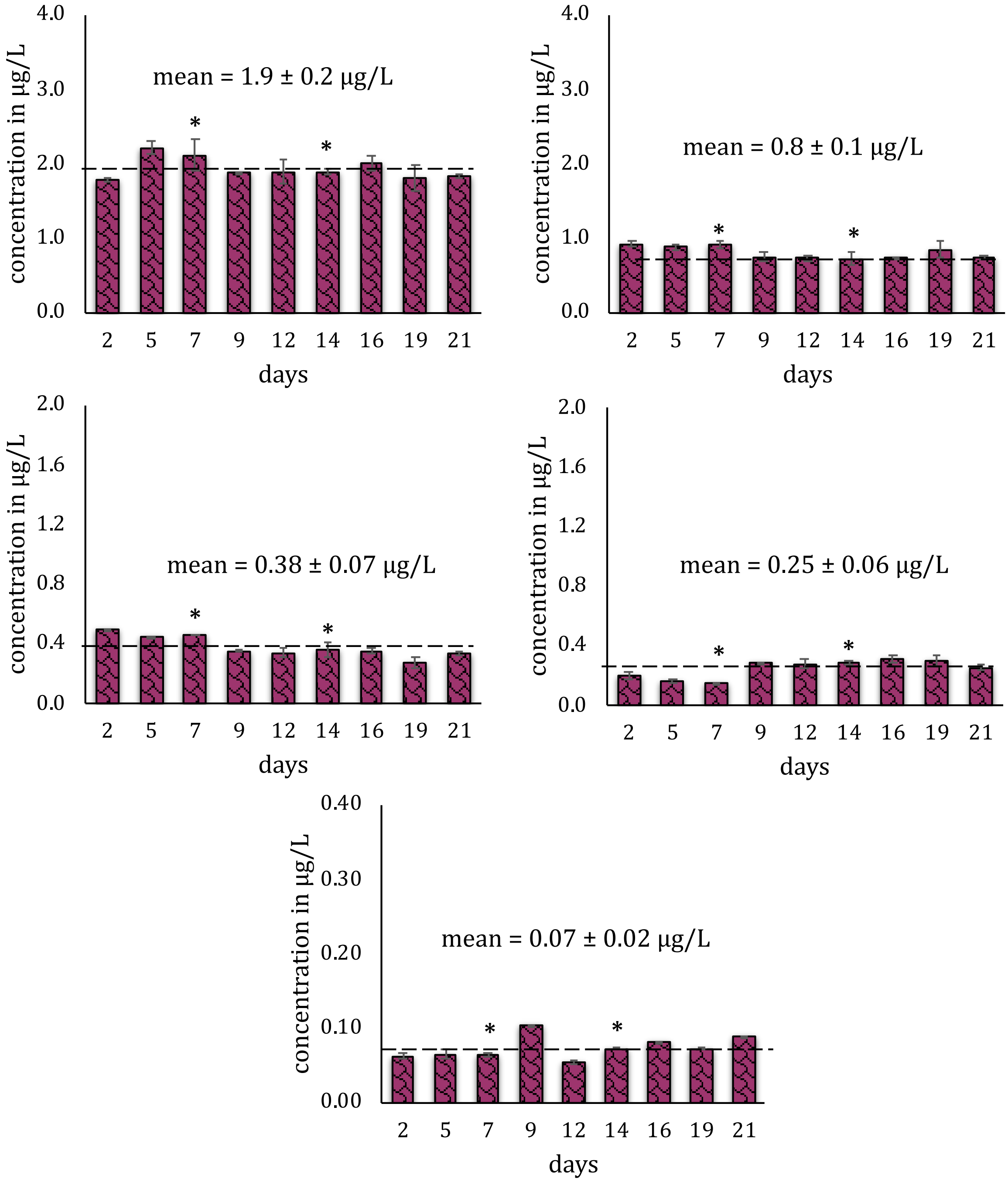


**Figure S5** Concentration monitoring of BNT during chronic toxicity tests. The polymers were freshly reloaded after each use. The average of all measured concentrations (shown with dashed lines) was taken to report the concentration. Bars marked with an asterisk indicate initial concentrations (after medium change, before adding animals and food), while the others indicate final concentrations (before medium change, after 2-3 days exposure to animals and food). For the two lowest exposure concentrations, i.e. 70 and 250 ng $L^{-1}$, replicates were pooled to have sufficient volume for extractions followed by chemical analysis.

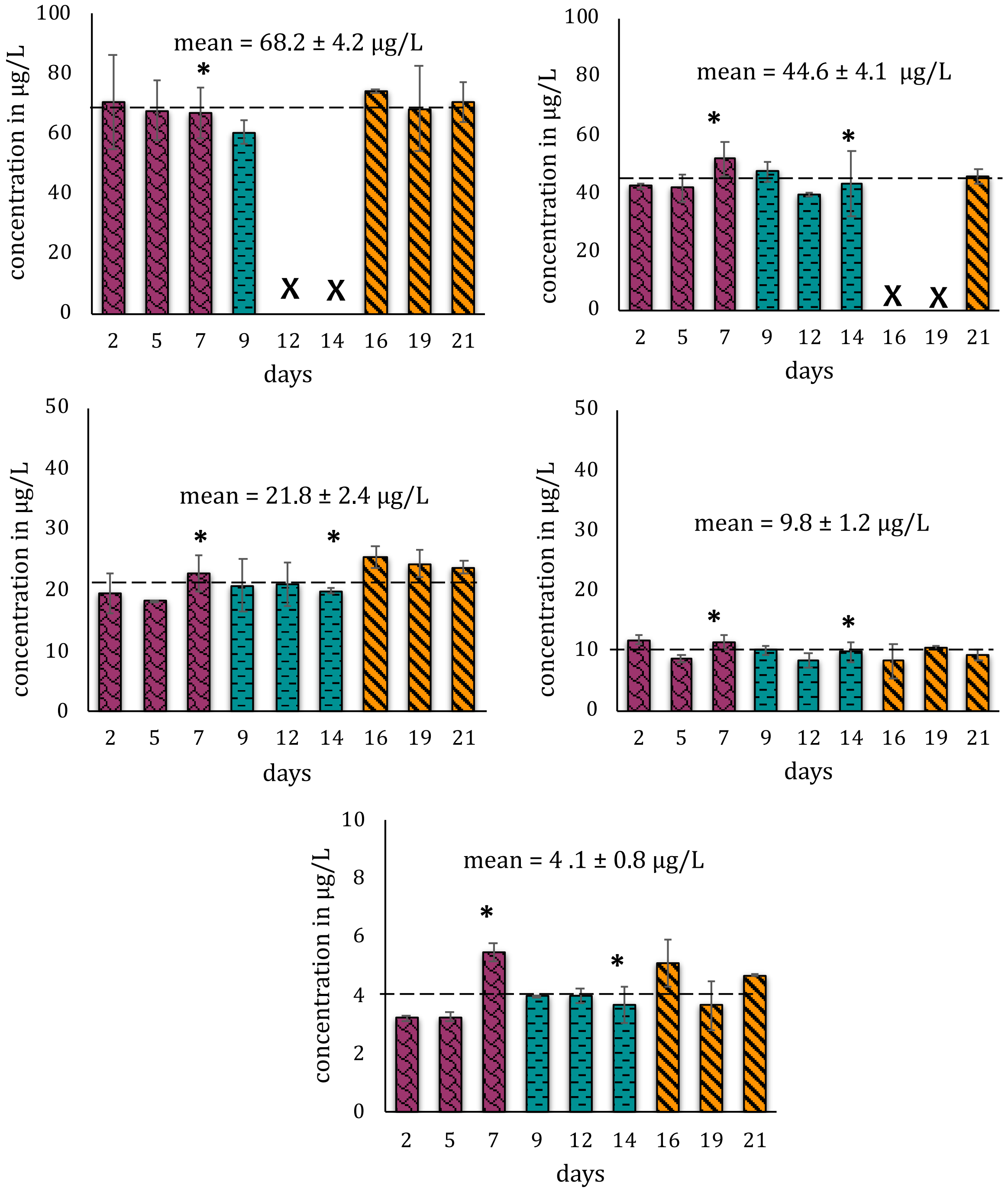


**Figure S6** Concentration monitoring of BCRB during chronic toxicity tests. The same polymers were used three times without reloading to dose the chemical into the exposure medium. Measured concentrations after each dosing cycle are shown in different colors, i.e., 1st cycle - purple, 2nd cycle - green, and 3rd cycle - orange. The average of all measured concentrations (shown with dashed lines) was taken to report the concentration. Bars marked with an asterisk indicate initial concentrations (after medium change, before adding animals and food), while the others indicate final concentrations (before medium change, after 2-3 days exposure to animals and food).

## S5. Concentration-response curve (CRC) fitting procedure

GraphPad Prism version 9.5.1 was used to fit CRC and obtain $EC_{10}$, $EC_{20}$, $EC_{50}$ values and their confidence intervals. The exposure concentrations were $\log_{10}$ transformed before fitting. Then a four parameters logistic model with variable slope was used that describes the response as a function of following four parameters: minimum response (the Bottom), maximum response (the Top), the concentration required to cause a response half-way between the minimum and maximum (the $EC_{50}$) and the slope of the curve (the HillSlope). The F represents effect level (i.e. 10% for $EC_{10}$), the Y is response and X is logarithm of concentration. The Top was constrained to 100, the Bottom was constrained to 0, the HillSlope was not constrained.

$$\log EC_{50} = \log ECF - \frac{1}{HillSlope} \times \log\left(\frac{F}{100 - F}\right)$$

$$Y = Bottom + \frac{Top - Bottom}{\left(1 + 10^{(\log EC_{50} - X) \times HillSlope}\right)}$$

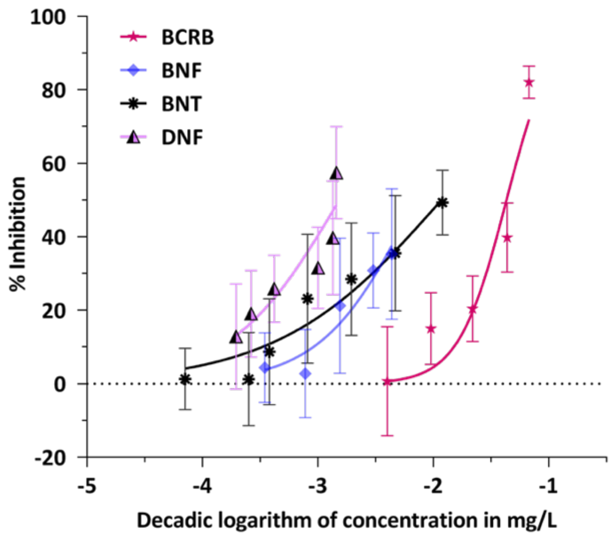


**Figure S7** Concentration-response curves of four NSO-PAHs based on reproductive inhibition during 21-day chronic toxicity tests with *D. magna*.

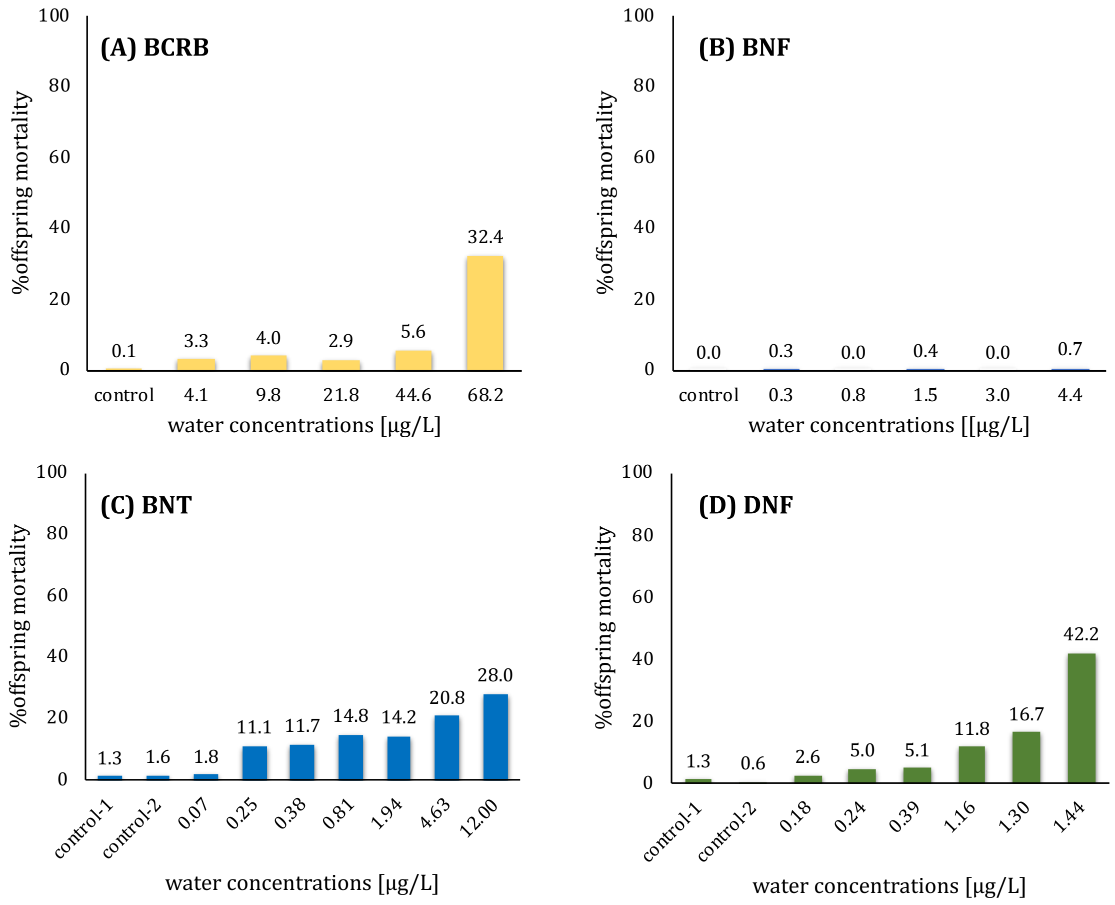


**Figure S8** Percentage of offspring mortality compared to the total number of offspring produced during 21-day chronic reproduction tests with *D. magna* after exposure to (**A**) BCRB, (**B**) BNF, (**C**) BNT, and (**D**) DNF at different concentration levels.

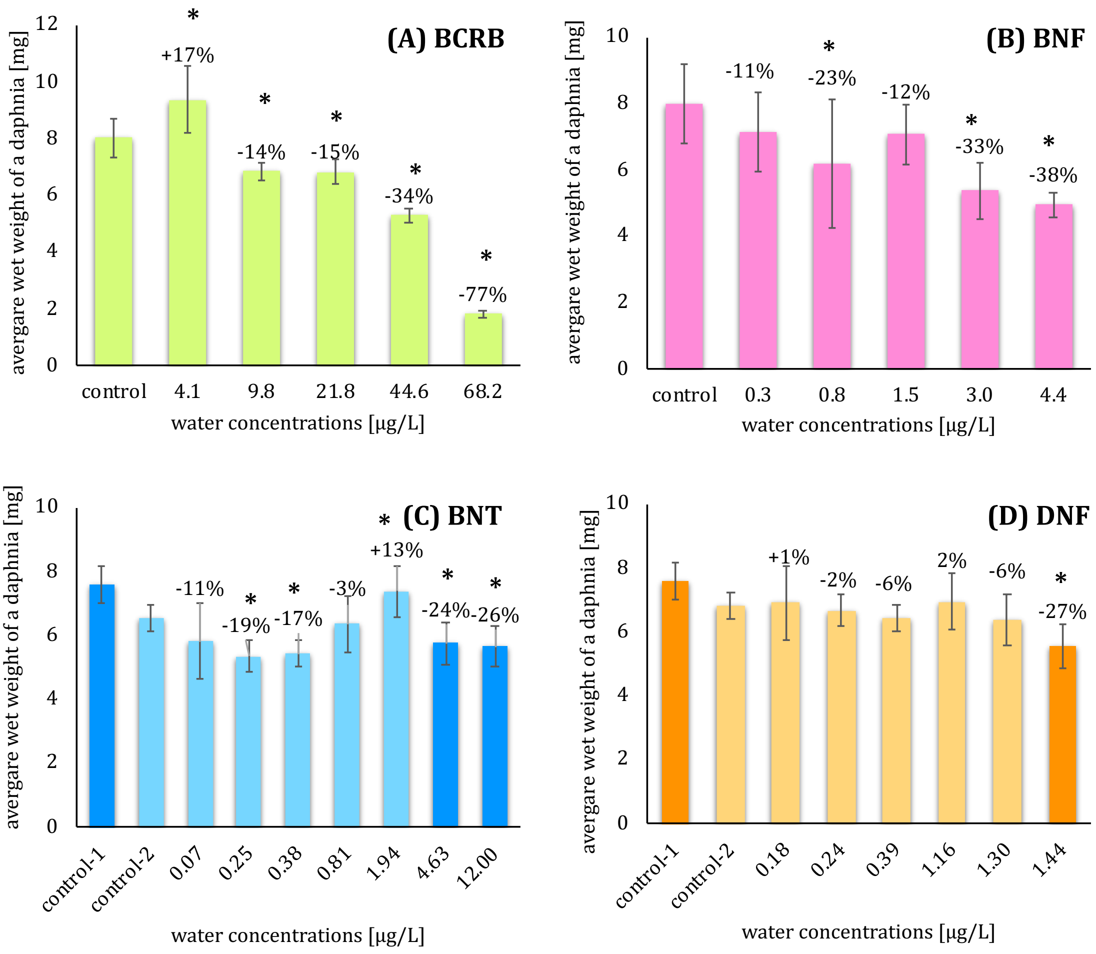


**Figure S9** Wet weight of parent daphnids at the end of 21-d exposure to (**A**) BCRB, (**B**) BNF, (**C**) BNT and (**D**) DNF at each concentration level (change relative to control indicated as ±% above the bars). Data for the latter two compounds (C and D) were obtained from two separate experiments, with the results from the same experiments shown with the same shades of blue (for C) or orange (for D). The weights of the test groups that are statistically significantly different (*p*-value < 0.05) from the controls are indicated by asterisks.

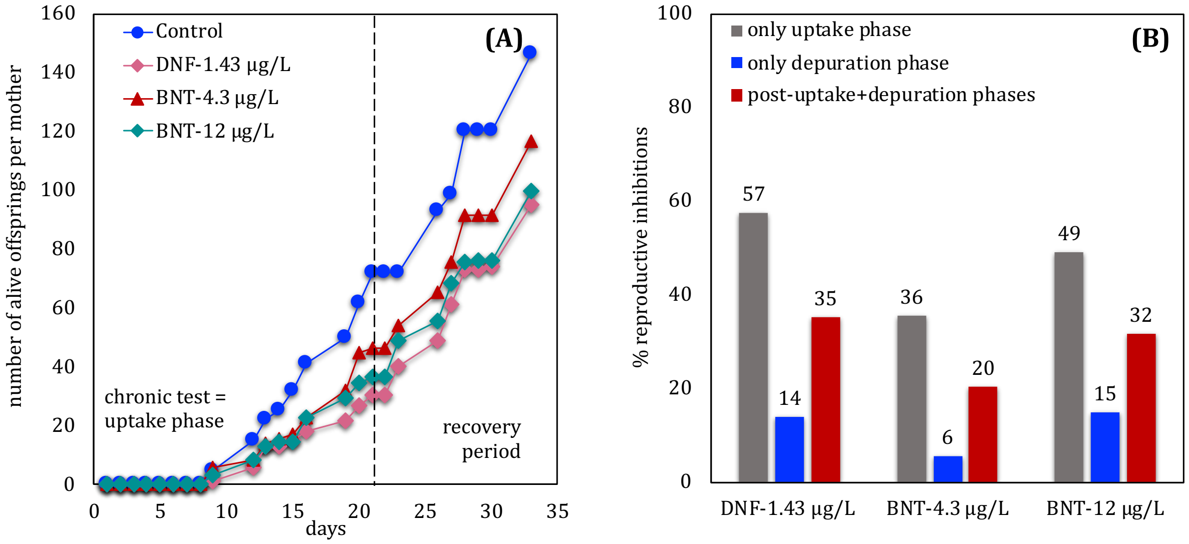


**Figure S10** Effect of DNF and BNT on reproduction of *D. magna* during combined bioaccumulation/chronic toxicity test. A 33-day test period consisting of a 21-day exposure period (served as uptake phase of the bioaccumulation test) and a 12-day recovery period (depuration phase of the bioaccumulation test) in clean medium. (**A**) the average number of offspring produced per mother in control and treatment groups, (**B**) the % reproductive inhibition during uptake phase only (days 0-21), depuration phase only (days 21-33), and entire test (days 1-33).

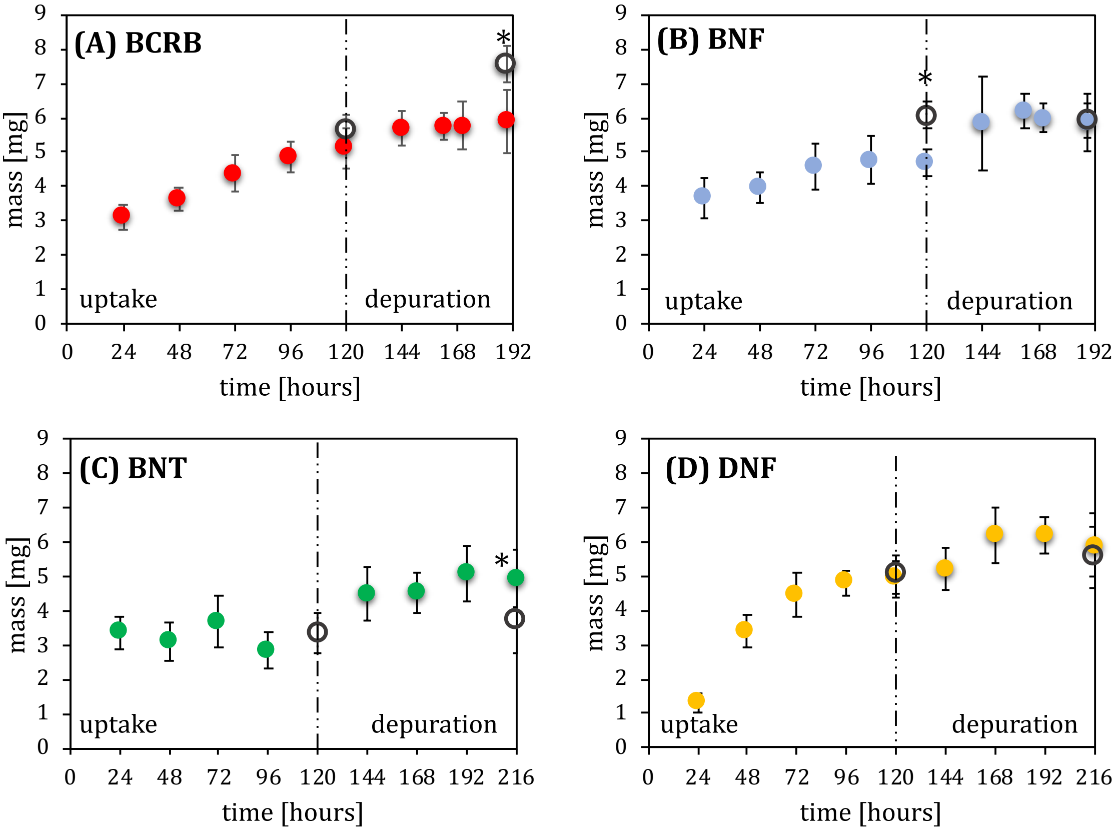


**Figure S11** Changes of body mass of daphnids (wet weight) during the uptake and depuration phases of bioaccumulation tests. Closed symbols - animals exposed to: (**A**) BCRB, (**B**) BNF, (**C**) BNT and (**D**) DNF (n=10-15); open black symbols - controls sampled only at the end of the uptake and depuration phases (n=5).

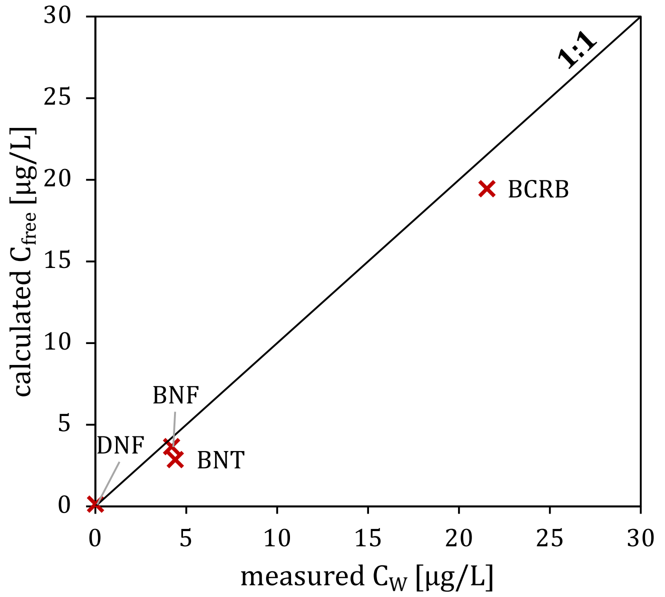


**Figure S12** Correlation between measured concentrations in medium ($C_W$) after centrifuging the biomass and the predicted freely dissolved concentrations ($C_{free}$) derived from partition coefficients between methanol and the medium ($K_{MeOH:medium}$). Corresponding $K_{MeOH:medium}$ values are given in Table S5.

## S6. Calculation of Partition Coefficient ($K_{MeOH:Medium}$)

The partition coefficient between the methanolic loading solution and the exposure medium ($K_{MeOH:Medium}$) was determined according to equation:

$$K_{MeOH:Medium} = \frac{C_{MeOH}}{C_{Medium}}$$

where:

$C_{MeOH}$ is the concentration of the chemical in the methanolic loading solution used to dose the PDMS disks.

$C_{Medium}$ is the equilibrium concentration of the chemical in the exposure medium after dosing with PDMS disks, measured before the introduction of animals and diet. This represents the freely dissolved concentration in the system, as no organic content was present.

The derived partition coefficients (Table S5) were used to determine the concentrations of methanolic loading required to achieve specific target concentrations in the exposure medium.

**Table S5** Partition coefficients between the methanolic loading solutions ($C_{MeOH,\ t=0,}$ nominal concentration in methanol at t=0) and the measured equilibrium concentrations of the aqueous medium ($C_{medium,\ eq}$).

| Compound | $C_{MeOH,\ t=0}$ [mg L$^{-1}$] | Measured $C_{medium,\ eq}$ [mg L$^{-1}$] | $K_{MeOH:medium}$ | log $K_{MeOH:medium}$ | log $K_{MeOH:medium}$ (mean ± SD) | Calculated $C_{medium,\ eq}$ [mg L$^{-1}$]* |
|---|---|---|---|---|---|---|
| | 1000 | 6.8E-02 | 1.5E+04 | 4.17 | | 7.59E-02 |
| | 500 | 4.3E-02 | 1.2E+04 | 4.06 | | 3.79E-02 |
| BCRB | 250 | 2.2E-02 | 1.1E+04 | 4.06 | 4.12±0.06 | 1.90E-02 |
| | 125 | 9.6E-03 | 1.3E+04 | 4.12 | | 9.48E-03 |
| | 63 | 4.0E-03 | 1.6E+04 | 4.20 | | 4.78E-03 |
| | 1000 | 4.4E-03 | 2.3E+05 | 5.36 | | 5.50E-03 |
| | 500 | 3.0E-03 | 1.7E+05 | 5.22 | | 2.75E-03 |
| BNF | 250 | 1.6E-03 | 1.6E+05 | 5.21 | 5.26±0.06 | 1.37E-03 |
| | 125 | 7.7E-04 | 1.6E+05 | 5.21 | | 6.87E-04 |
| | 63 | 3.5E-04 | 1.8E+05 | 5.26 | | 3.46E-04 |
| | 2500 | 1.2E-02 | 2.1E+05 | 5.32 | | 8.67E-03 |
| | 1000 | 4.7E-03 | 2.1E+05 | 5.33 | | 3.47E-03 |
| | 500 | 1.9E-03 | 2.6E+05 | 5.41 | | 1.73E-03 |
| BNT | 250 | 8.1E-04 | 3.1E+05 | 5.49 | 5.46±0.12 | 8.67E-04 |
| | 125 | 3.8E-04 | 3.3E+05 | 5.52 | | 4.33E-04 |
| | 63 | 2.5E-04 | 2.5E+05 | 5.40 | | 2.18E-04 |
| | 31 | 7.0E-05 | 4.4E+05 | 5.65 | | 1.07E-04 |
| | saturated | 1.4E-03 | - | - | | - |
| | 1500 | 1.3E-03 | 1.2E+06 | 6.06 | | 1.57E-03 |
| | 1000 | 1.2E-03 | 8.0E+05 | 5.91 | | 1.05E-03 |
| DNF | 500 | 4.1E-04 | 1.2E+06 | 6.08 | 5.98±0.14 | 5.24E-04 |
| | 250 | 2.6E-04 | 9.5E+05 | 5.98 | | 2.62E-04 |
| | 125 | 2.0E-04 | 6.4E+05 | 5.81 | | 1.31E-04 |

* The "calculated $C_{Medium,\ eq}$" were reported using the average $K_{MeoH:medium}$ and $C_{MeOH,\ t=0}$.

## S7. Quality Control Measures in Bioaccumulation Tests

In bioaccumulation tests, the test animals were genetically identical copies of the mother; therefore, we expect relatively low variability in the population. Nevertheless, to account for individual variability and to obtain sufficient biomass for analysis while keeping the experimental effort manageable, two replicates of five individuals each (pooled) were analysed at each time point. Background contamination was controlled by quantifying chemicals in control animals and their media. To minimize bias, animals were randomly sampled at each time point and standardized handling, sampling, and analysis protocols were followed. Exposure variability was reduced by using passive dosing.

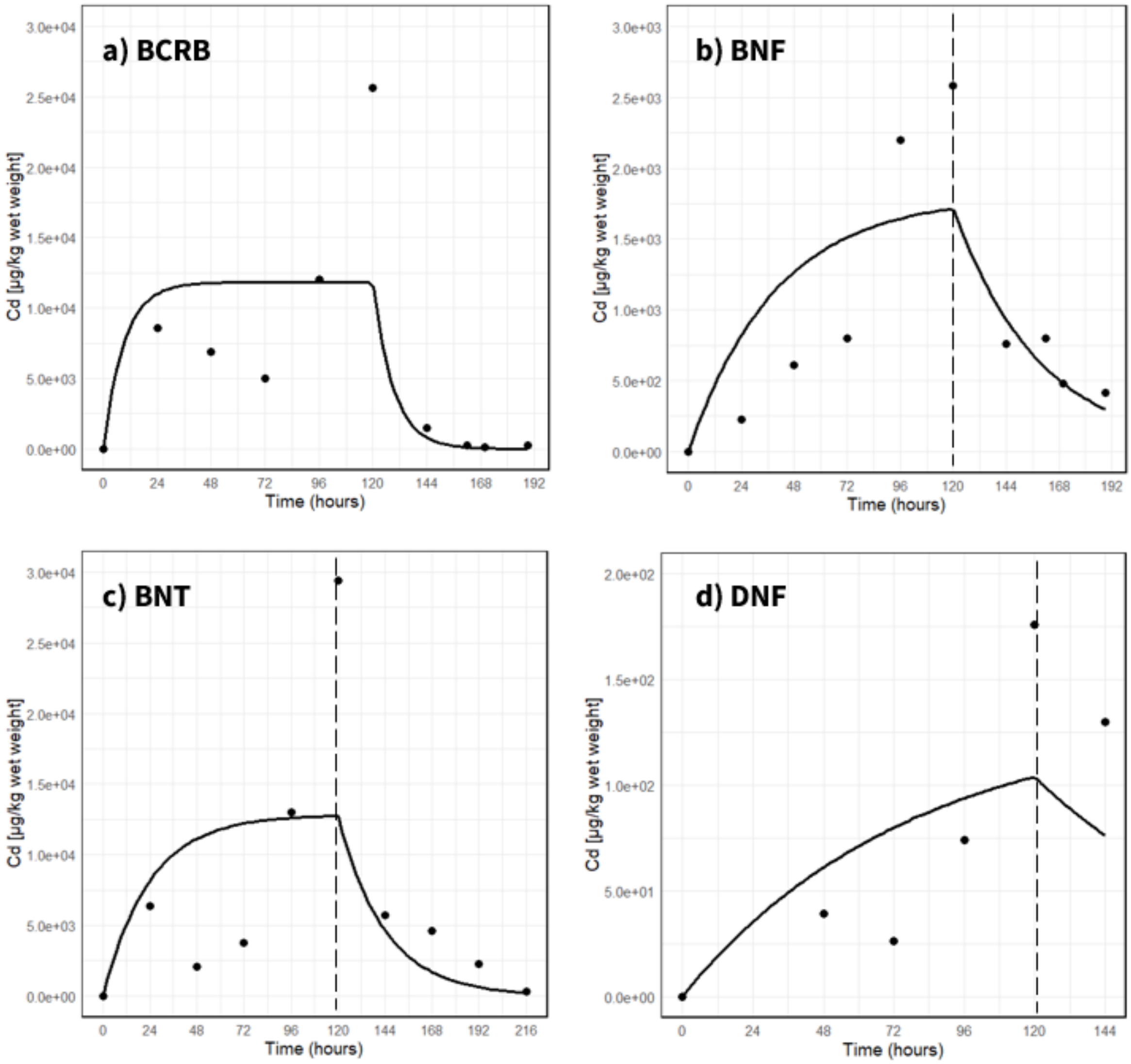


**Figure S13** Uptake and depuration kinetics of four heterocyclic PAHs in *D. magna* via water and diet exposure. The uptake phase was completed after 120 hours and was followed by the depuration phase. Solid lines shows the fitted models using the equations[3] : (1) $C_D = C_W \cdot \frac{k_1}{k_2} \cdot (1 - e^{-k_2 t})$ when $0 < t < t_c$ where $t_c$ is the time at the end of the uptake phase, and (2) $C_D = C_W \cdot \frac{k_1}{k_2} \cdot (e^{-k_2(t-t_c)} - e^{-k_2 t})$ when $t > t_c$ and black points represent measured concentrations.

**Table S6** The $BCF_{L5\%}$ values of organic compounds in *Daphnia* (*D. magna* or *D. pulex*) and in fish (dataset used in Fig. 3B).

| | | ***D. magna or D. pulex*** | | | **fish** | | |
|---|---|---|---|---|---|---|---|
| **Compound** | **log $K_{OW}$** | **BCF** | **log $BCF_{L5\%}$** | **Ref.** | **BCF** | **log $BCF_{L5\%}$** | **Ref.** |
| naphthalene | 3.30 | 131 | 2.64 | [4] | 26 | 2.71 | [4] |
| fluorene | 4.18 | 506 | 3.23 | [4] | 83 | 3.22 | [4] |
| anthracene | 4.45 | 970; 917 (M=944) | 3.50 | [4] | 2231 | 4.65 | [4] |
| phenanthrene | 4.46 | 324;325 (M=325) | 3.03 | [4] | 236 | 3.68 | [4] |
| fluoranthene | 5.81 | 1742 | 3.76 | [4] | 137 | 3.44 | [4] |
| pyrene | 4.88 | 2702; 2200 (M=2451) | 3.91 | [4,5] | 74 | 3.17 | [4] |
| benz[a]anthracene | 5.76 | 10226; 10109 (M=10168) | 4.53 | [4] | 260 | 3.72 | [4] |
| benzo[a]pyrene | 6.13 | 12761; 2837 (M=7799) | 4.41 | [4] | 367; 608; 30 (M=367) | 3.87 | [4] |
| quinoline | 2.05 | 3 | 1.00 | [6] | 2; 3 (M=2.5) * | 1.92 | [7] |
| carbazole | 3.72 | 115 | 2.58 | [8] | 500 * | 2.44 | [7] |
| dibenzothiophene | 4.65 | 600 | 3.30 | [9] | 6620 * | 3.57 | [7] |
| benzonapthothiophene | 5.75 | 8000 | 4.43 | [9] | 14900 * | 3.92 | [7] |
| dibenzocarbazole | 6.40 | 7126 | 4.38 | [8] | 7060 * | 3.59 | [7] |
| acridine | 3.41 | 30 | 2.00 | [10] | 1300 * | 2.86 | [7] |
| hexachlorobenzene | 5.73 | 910 | 3.48 | [11] | 2700; 4800; 1600; 3900; 11000; 27000; 6000; 30000 (M=5400) | 5.03 | [12] |
| DDT | 6.19 | 18540 | 4.79 | [13] | 44668 | 5.95 | [14] |
| pp'DDE | 6.51 | 28600 | 4.98 | [12] | 27500; 81000 (M=54250) | 6.04 | [12] |
| terbutryn | 3.74 | 15 | 1.70 | [13] | 17 | 2.53 | [15] |
| atrazine | 2.75 | 2 | 0.82 | [13] | 2 | 1.60 | [15] |
| metolachlor | 3.13 | 5 | 1.22 | [13] | 7 | 2.15 | [15] |
| monuron | 2.08 | 2 | 0.82 | [13] | 2 | 1.60 | [15] |
| thiazafluoron | 1.85 | 2 | 0.82 | [13] | 1 | 1.30 | [15] |
| chrysene | 6.40 | 5500 | 4.26 | [13] | 6088 | 5.09 | [13] |

The actual relative lipid weight ($f_{lipid}$) is assumed to be 1.5% in *Daphnia magna* and 5% in fish, except for data points marked with an "*" where the relative lipid content of fish is reported as 9% in the original study. All log $BCF_{L5\%}$ values are then normalized to a 5% lipid content to facilitate comparisons, using the equation: log $BCF_{L5\%}$ = (BCF x 0.05 / $f_{lipid}$).

**Table S7** Bioaccumulation data (log $BCF_{L5\%}$, $k_2$, $t_{50}$) for *D. magna or D. pulex* (dataset used in Fig. 3A and Fig. 4).

| Chemical | log $K_{OW}$ | log $k_2$ [$h^{-1}$] | log $BCF_{L5\%}$ | half-life [h] | Ref |
|---|---|---|---|---|---|
| isoquinoline | 1.82 | 1.26 | 0.9 | 0.04 | [10] |
| acridine | 3.3 | 0.33 | 2 | 0.32 | [10] |
| naphthalene | 3.3 | 0.22 | 2.64 | 0.42 | [10] |
| carbazole | 3.72 | 0.31 | 2.58 | 0.34 | [8] |
| benz(a)acridine | 4.45 | -0.24 | 3.07 | 1.2 | [10] |
| phenanthrene | 4.45 | -0.27 | 3.03 | 1.29 | [10] |
| anthracene | 4.45 | -0.23 | 3.48 | 1.18 | [10] |
| pyrene | 4.9 | -0.46 | 3.95 | 2 | [10] |
| BCRB | 5.22 | -0.96* | 3.6 | 6.23 | this study |
| 9-methyl-anthracene | 5.56 | -0.84 | 4.18 | 4.8 | [10] |
| benz(a)anthracene | 5.6 | -0.84 | 4.52 | 4.8 | [10] |
| BNF † | 5.6 | -1.66* | 3.28† | 27.73 | this study |
| BNT | 5.85 | -1.39* | 4.36 | 16.5 | this study |
| perylene | 6.06 | -0.86 | 4.37 | 5.02 | [10] |
| dibenzocarbazole | 6.4 | -1.11 | 4.38 | 9 | [8] |
| DNF † | 6.89 | -1.98* | 3.77† | 53.73 | this study |
| thiazafluoron | 1.85 | n.a. | 0.9 | n.a. | [13] |
| quinoline | 2.05 | n.a. | 0.95 | n.a. | [6] |
| monuron | 2.08 | n.a. | 0.84 | n.a. | [13] |
| atrazine | 2.75 | n.a. | 0.78 | n.a. | [13] |
| metolachlor | 3.13 | n.a. | 1.25 | n.a. | [13] |
| fluorodifen | 3.3 | n.a. | 1.7 | n.a. | [13] |
| pentachlorophenol | 3.69 | n.a. | 2.56 | n.a. | [13] |
| lindane | 3.72 | n.a. | 2.86 | n.a. | [13] |
| terbutryn | 3.74 | n.a. | 1.69 | n.a. | [13] |
| 1,2,4-trichlorobenzene | 4.05 | n.a. | 2.67 | n.a. | [13] |
| fluorene | 4.18 | n.a. | 3.22 | n.a. | [4] |
| dibenzothiophene | 4.65 | n.a. | 3.3 | n.a. | [9] |
| benzanthrone | 4.81 | n.a. | 3.24 | n.a. | [16] |
| benzo[a]fluorene | 5.4 | n.a. | 4.09 | n.a. | [16] |
| triphenylene | 5.45 | n.a. | 4.48 | n.a. | [16] |
| hexachlorobenzene | 5.73 | n.a. | 3.48 | n.a. | [11] |
| benzo[b]fluorene | 5.75 | n.a. | 4.41 | n.a. | [16] |
| benzonapthothiophene | 5.75 | n.a. | 4.42 | n.a. | [9] |
| benzo[b]anthracene | 5.76 | n.a. | 4.52 | n.a. | [16] |
| chrysene | 5.81 | n.a. | 4.31 | n.a. | [16] |
| fluoranthene | 5.81 | n.a. | 3.76 | n.a. | [4] |
| benzo[k]fluoranthrene | 6.11 | n.a. | 4.64 | n.a. | [16] |
| benzo[a]pyrene | 6.13 | n.a. | 4.41 | n.a. | [4] |
| DDT | 6.19 | n.a. | 4.79 | n.a. | [13] |

| Chemical | log $K_{OW}$ | log $k_2$ [$h^{-1}$] | log $BCF_{L5\%}$ | half-life [h] | Ref |
|---|---|---|---|---|---|
| benzo[g,h,i]perylene | 6.2 | n.a. | 4.97 | n.a. | 16 |
| perylene | 6.3 | n.a. | 4.38 | n.a. | 16 |
| benzo[e]pyrene | 6.44 | n.a. | 4.92 | n.a. | 16 |
| pp'DDE | 6.51 | n.a. | 4.98 | n.a. | 12 |
| dibenz[ah]anthracene | 6.84 | n.a. | 5.22 | n.a. | 16 |

*The growth-dilution corrected $k_2$ values were reported for NSO-PAHs i.e. $k_{2g}$. †BNF and DNF are shown but excluded from the correlation in Fig. 4 due to the fact that the $BAF^*_{L5\%}$ is probably considerably underestimated ($t_{95}$ equal to or higher than the duration of uptake phase).

**Table S8** Bioaccumulation data (log $BCF_{L5\%}$, $k_2$, $t_{50}$) for *H. azteca* (dataset used in Fig. 3A and Fig. 4.)

| Chemical | log $K_{OW}$ | log $k_2$ [$h^{-1}$] | log $BCF_{L5\%}$ | half-life [h] | Ref |
|---|---|---|---|---|---|
| Hexachlorobenzene | 5.86 | -1.76 | 4.41 | 39.89 | 17 |
| Ortho-terphenyl | 5.52 | -1.71 | 4.01 | 35.78 | 17 |
| Methoxychlor | 5.67 | -1.95 | 4.19 | 61.39 | 17 |
| 1,2,3-trichlorobenzene | 3.93 | -1.40 | 1.66 | 17.27 | 17 |
| 2,4,5-trichlorphenol | 3.45 | -1.41 | 2.46 | 17.62 | 17 |
| PCB153 | 7.62 | -2.48 | 5.41 | 210.58 | 17 |
| PCB77 | 6.34 | -2.17 | 5.01 | 101.44 | 17 |
| Diazinon | 3.86 | -1.20 | 1.91 | 10.94 | 17 |
| Chlorpyrifos | 4.66 | -1.71 | 3.29 | 35.17 | 17 |
| 14C methoxychlor | 5.67 | -1.48 | 3.79 | 20.85 | 17 |
| 14C pyrene | 4.93 | -1.53 | 3.77 | 23.30 | 17 |
| UV-234 | 7.67* | -1.94 | 3.20 | 60.94 | 18 |
| UV-329 | 6.35* | -1.56 | 4.30 | 25.32 | 18 |
| terbutryn | 3.60 | -0.44 | 1.77 | 1.93 | 19 |
| prochloraz | 4.40 | -0.67 | 2.19 | 3.27 | 19 |
| azoxystrobin | 2.50 | 0.08 | 0.73 | 0.58 | 20 |
| diclofenac | 0.70 | 0.39 | 0.35 | 0.28 | 20 |
| trifloxystrobin | 4.50 | 0.20 | 2.93 | 0.44 | 20 |
| fluorene | 3.22 | -0.01 | 2.68 | 0.72 | 21 |
| phenantrene | 4.46 | -0.50 | 2.89 | 2.18 | 21 |
| pyrene | 4.88 | -0.84 | 3.80 | 4.75 | 21 |
| PCP | 4.45 | -1.17 | 2.34 | 10.19 | 22 |
| methyl parathion | 2.04 | -1.82 | 1.30 | 46.20 | 22 |
| fluoranthene | 5.22 | -1.59 | 3.54 | 26.65 | 22 |
| HCBP | 6.65 | -2.40 | 5.07 | 173.25 | 22 |

*The log $K_{OW}$ values for UV-234 and UV-329 were predicted by the KOWWIN model (EPI Suite™).

**Table S9** Bioaccumulation data (log $BCF_{L5\%}$) for *H. Azteca* and fish (dataset used in Fig. 3B)

| Chemical | log $K_{OW}$ | *H. azteca* log $BCF_{L5\%}$ | Ref | Fish log $BCF_{L5\%}$ | Ref |
|---|---|---|---|---|---|
| Hexachlorobenzene | 5.86 | 4.41 | 17 | 5.03 | 12 |
| Ortho-terphenyl | 5.52 | 4.01 | 17 | 3.40 | 17 |
| Methoxychlor | 5.67 | 4.19 | 17 | 3.20 | 17 |
| 1,2,3-trichlorobenzene | 3.93 | 1.66 | 17 | 2.40 | 17 |
| 2,4,5-trichlorphenol | 3.45 | 2.46 | 17 | 2.60 | 17 |
| PCB153 | 7.62 | 5.41 | 17 | 5.20 | 17 |
| PCB77 | 6.34 | 5.01 | 17 | 5.00 | 17 |
| Diazinon | 3.86 | 1.91 | 17 | 1.80 | 17 |
| Chlorpyrifos | 4.66 | 3.29 | 17 | 2.70 | 17 |
| 14C pyrene | 4.93 | 3.77 | 17 | 2.70 | 17 |
| UV-234 | 7.67 | 3.18 | 18 | 3.00 | 18 |
| UV-329 | 6.35 | 4.30 | 18 | 2.60 | 18 |
| Benzo(a)pyrene | 6.11 | 3.81 | 17 | 3.87 | 4 |
| 14C LHC | 3.36 | 2.95 | 17 | 1.90 | 17 |
| 14C simazine | 2.40 | 1.18 | 17 | 1.10 | 17 |
| terbutryn | 3.60 | 1.77 | 19 | 2.53 | 15 |
| fluorene | 3.22 | 2.68 | 21 | 3.22 | 4 |
| phenantrene | 4.46 | 2.89 | 21 | 3.68 | 4 |
| pyrene | 4.88 | 3.80 | 21 | 3.17 | 4 |
| fluoranthene | 5.22 | 3.54 | 22 | 3.44 | 4 |

**Table S10** Fish bioaccumulation data (log $BCF_{L5\%}$, $k_2$, $t_{50}$) from UK Environment Agency Report[23] (dataset used in Fig. 3A and Fig. 4.)

| Chemical | log $K_{OW}$ | log $k_2$ [$h^{-1}$] | log $BCF_{L5\%}$ | half-life [h] |
|---|---|---|---|---|
| C-12-2-LAS | 4.71 | -1.54 | 2.16 | 23.77 |
| Octaethylene glycol monotridecyl ether | 3.07 | -0.38 | 1.69 | 1.65 |
| Octaethylene glycol monotridecyl ether | 5.11 | -0.38 | 1.69 | 1.65 |
| C-12-5-LAS | 4.71 | -1.30 | 1.00 | 13.86 |
| NL-63A | 6.90 | -2.12 | 4.04 | 92.42 |
| NL-63B | 6.85 | -2.18 | 3.83 | 103.97 |
| Methyl isocyanothion | 3.58 | -1.31 | 2.09 | 14.10 |
| *O,O*-Dimethyl *O*-[3-methyl-4-(methylthio)phenyl]ester phosphorothioic acid | 4.17 | -1.60 | 2.92 | 27.73 |
| *O,O*-Dimethyl *O*-[3-methyl-4-(methylthio)phenyl]ester phosphorothioic acid | 4.17 | -0.70 | 2.21 | 3.47 |
| *O,O*-Dimethyl *O*-[3-methyl-4-(methylthio)phenyl]ester phosphorothioic acid | 4.17 | -0.47 | 1.43 | 2.04 |
| *O,O*-Dimethyl *O*-[3-methyl-4-(methylthio)phenyl]ester phosphorothioic acid | 4.17 | -0.85 | 2.02 | 4.95 |
| Benzo[a]anthracene | 5.76 | -1.20 | 2.43 | 10.87 |
| Cyclohexane, 1,2,3,4,5,6- hexachloro-, (1a,2a,3B,4a,5a,6B)- | 3.72 | -1.26 | 2.81 | 12.60 |

| Chemical | log $K_{OW}$ | log $k_2$ [$h^{-1}$] | log $BCF_{L5\%}$ | half-life [h] |
|---|---|---|---|---|
| Cyclohexane, 1,2,3,4,5,6- hexachloro-, (1a,2a,3B,4a,5a,6B)- | 3.72 | -1.63 | 2.83 | 29.71 |
| Cyclohexane, 1,2,3,4,5,6- hexachloro-, (1a,2a,3B,4a,5a,6B)- | 3.72 | -1.52 | 2.81 | 23.10 |
| Cyclohexane, 1,2,3,4,5,6- hexachloro-, (1a,2a,3B,4a,5a,6B)- | 3.72 | -1.42 | 2.82 | 18.08 |
| Cyclohexane, 1,2,3,4,5,6- hexachloro-, (1a,2a,3B,4a,5a,6B)- | 3.72 | -1.82 | 2.81 | 46.21 |
| Benzenamine | 0.90 | 0.81 | 0.32 | 0.11 |
| Phenol, 4,4 -(1- methylethylidene)bis- | 3.32 | -0.20 | 0.80 | 1.10 |
| Benzene, 1,2,3-trichloro- | 4.05 | -1.75 | 2.46 | 38.69 |
| Phenol, pentachloro- | 5.12 | -1.60 | 2.15 | 27.73 |
| Phenol, pentachloro- | 5.12 | -1.97 | 2.52 | 63.98 |
| Phenol, pentachloro- | 5.12 | -1.37 | 1.91 | 16.15 |
| Phenol, pentachloro- | 5.12 | -1.19 | 2.36 | 10.66 |
| Phenol, pentachloro- | 5.12 | -0.78 | 2.13 | 4.22 |
| Phenol, pentachloro- | 5.12 | -1.19 | 2.80 | 10.66 |
| 2,4,6-Trichlorophenol | 3.69 | -0.70 | 1.55 | 3.48 |
| 4,4'-Dibromo biphenyl | 5.72 | -2.30 | 4.13 | 138.63 |
| Benzenamine, 2-chloro- | 1.90 | -0.72 | 0.32 | 3.65 |
| Benzenamine, 2-chloro- | 1.90 | -0.72 | 0.62 | 3.65 |
| Benzene, 1,2,4,5-tetrachloro- | 4.64 | -1.78 | 3.38 | 41.59 |
| 4-Chlorobenzaldehyde | 2.10 | -0.96 | 0.32 | 6.30 |
| Benzene, 1,4-dibromo- | 3.79 | -1.23 | 1.85 | 11.80 |
| Benzene, 1,4-dichloro- | 3.44 | -1.39 | 2.24 | 16.98 |
| Benzenamine, 4-chloro- | 1.83 | 0.62 | 0.86 | 0.17 |
| Benzenamine, 4-chloro- | 1.83 | -0.80 | 0.02 | 4.33 |
| Benzenamine, 4-chloro- | 1.83 | -0.80 | 0.32 | 4.33 |
| Benzenamine, 3-chloro- | 1.88 | -0.68 | 0.02 | 3.30 |
| Benzenamine, 3-chloro- | 1.88 | -0.68 | 0.32 | 3.30 |
| Phosphoric acid, triphenyl ester | 4.59 | -0.24 | 2.30 | 1.20 |
| Ethanol, 2-chloro-, phosphate (3:1) | 1.44 | 0.00 | 0.02 | 0.70 |
| 1,2,4,5-Tetrachloro-3- nitrobenzene | 4.38 | -1.93 | 2.91 | 59.41 |
| 1,2-Benzenedicarboxylic acid, bis(2-ethylhexyl) ester | 7.73 | -2.69 | 2.79 | 339.50 |
| 1,2-Benzenedicarboxylic acid, bis(2-ethylhexyl) ester | 7.73 | -2.34 | 2.81 | 151.23 |
| 1,2-Benzenedicarboxylic acid, bis(2-ethylhexyl) ester | 7.73 | -2.61 | 2.99 | 281.96 |
| 1,2-Benzenedicarboxylic acid, bis(2-ethylhexyl) ester | 7.73 | -2.34 | 3.02 | 151.23 |
| Benzene, 2-methyl-1,3,5-trinitro- | 1.60 | 0.01 | 1.02 | 0.68 |
| Phosphorothioic acid, *O*-(1,6- dihydro-6-oxo-1-phenyl-3- pyridazinyl) *O*,*O*-diethyl ester | 3.20 | -0.37 | 0.72 | 1.62 |
| Benzene, 1,2,4-trichloro- | 4.02 | -1.62 | 2.95 | 29.19 |
| Benzene, 1-methyl-2,4-dinitro- | 1.98 | -0.30 | 0.33 | 1.40 |
| Benzene, 1-methyl-2,4-dinitro- | 1.98 | -1.25 | 0.68 | 12.32 |

| Chemical | log $K_{OW}$ | log $k_2$ [$h^{-1}$] | log $BCF_{L5\%}$ | half-life [h] |
|---|---|---|---|---|
| Butanedioic acid, [(dimethoxyphosphinothioyl)thio]-, diethylester | 2.36 | -0.57 | 1.06 | 2.57 |
| 1,3,5-Triazine, hexahydro-1,3,5- trinitro- (RDX) | 0.87 | -0.20 | 0.32 | 1.09 |
| 1,3,5-Triazine, hexahydro-1,3,5- trinitro- (RDX) | 0.87 | -1.06 | 0.32 | 7.88 |
| *O*,*O*-Dimethyl *O*-(3-methyl-4- nitrophenyl) ester phosphorothioic acid (Fenitrothion) | 3.47 | -1.33 | 2.06 | 14.72 |
| *O*,*O*-Dimethyl *O*-(3-methyl-4- nitrophenyl) ester phosphorothioic acid (Fenitrothion) | 3.47 | -0.95 | 1.74 | 6.23 |
| *O*,*O*-Dimethyl *O*-(3-methyl-4- nitrophenyl) ester phosphorothioic acid (Fenitrothion) | 3.47 | -1.09 | 2.46 | 8.62 |
| *O*,*O*-Dimethyl *O*-(3-methyl-4- nitrophenyl) ester phosphorothioic acid (Fenitrothion) | 3.47 | -0.92 | 2.38 | 5.76 |
| *O*,*O*-Dimethyl *O*-(3-methyl-4- nitrophenyl) ester phosphorothioic acid (Fenitrothion) | 3.47 | -0.94 | 2.50 | 6.01 |
| *O*,*O*-Dimethyl *O*-(3-methyl-4- nitrophenyl) ester phosphorothioic acid (Fenitrothion) | 3.47 | -0.95 | 2.32 | 6.23 |
| *O*,*O*-Dimethyl *O*-(3-methyl-4- nitrophenyl) ester phosphorothioic acid (Fenitrothion) | 3.47 | -0.96 | 2.03 | 6.30 |
| *O*,*O*-Dimethyl *O*-(3-methyl-4- nitrophenyl) ester phosphorothioic acid (Fenitrothion) | 3.47 | -0.77 | 1.70 | 4.08 |
| Simazine | 2.18 | -0.11 | 0.62 | 0.90 |
| Phosphoric acid tributyl ester | 4.00 | -0.26 | 1.49 | 1.25 |
| 1H-Isoindole-1,3(2H)-dione, 3a,4,7,7a-tetrahydro-2-[(trichloro methyl)thio]- | 2.80 | -2.00 | 2.00 | 69.31 |
| 1H-Isoindole-1,3(2H)-dione, 3a,4,7,7a-tetrahydro-2-[(trichloro methyl)thio]- | 2.80 | -1.70 | 2.58 | 34.66 |
| Phenol, 4-(1,1,3,3-tetramethyl butyl)- | 5.28 | -1.05 | 2.77 | 7.70 |
| Dibenz(a,h) acridine | 5.67 | -0.85 | 2.05 | 4.95 |
| Phosphorothioic acid, *O*,*O*- dimethyl *O*-(4-nitrophenyl) ester | 3.04 | -1.00 | 1.68 | 6.99 |
| Phosphorothioic acid, *O*,*O*- dimethyl *O*-(2,4,5-trichlorophenyl)ester | 5.07 | -1.80 | 3.34 | 43.78 |
| (1alpha,2alpha,3beta,4alpha,5 beta,6beta) 1,2,3,4,5,6- Hexachloro cyclohexane | 3.80 | -1.34 | 2.92 | 15.26 |
| (1alpha,2beta, 3alpha,4beta, 5alpha,6beta)-1,2,3,4,5,6- Hexachloro-cyclohexane | 3.80 | -1.55 | 3.04 | 24.83 |
| (1alpha,2alpha,3alpha,4beta, 5alpha,6 beta)-1,2,3,4,5,6- hexachlorocyclohexane | 4.14 | -1.48 | 3.13 | 20.79 |
| Phosphorothioic acid, *O*,*O*- diethyl *O*-[6-methyl-2-(1- methylethyl)-4-pyrimidinyl] ester | 3.81 | -1.10 | 1.79 | 8.66 |
| Phosphorothioic acid, *O*,*O*- diethyl *O*-[6-methyl-2-(1- methylethyl)-4-pyrimidinyl] ester | 3.81 | -1.52 | 2.29 | 23.10 |
| Phosphorothioic acid, *O*,*O*- diethyl *O*-[6-methyl-2-(1- methylethyl)-4-pyrimidinyl] ester | 3.81 | -0.92 | 1.70 | 5.78 |
| Phosphorothioic acid, *O*,*O*- diethyl *O*-[6-methyl-2-(1-methylethyl)-4-pyrimidinyl] ester | 3.81 | -0.68 | 1.42 | 3.30 |
| Phosphorothioic acid, *O*-(3- Chloro-4-nitrophenyl) *O*,*O*- dimethyl ester | 3.63 | -0.54 | 1.31 | 2.38 |
| Octamethyl cyclotetra siloxane (D4) | 5.10 | -2.12 | 3.98 | 92.42 |
| Benzene, 1,3,5-tribromo- | 4.51 | -1.76 | 3.12 | 39.61 |
| 2,3,4-Trichloroaniline | 3.33 | -0.70 | 1.56 | 3.47 |
| 3,4,5-Trichloroaniline | 3.32 | -0.89 | 1.92 | 5.33 |
| 2,4,6-Trichloroaniline | 3.52 | -1.54 | 1.89 | 23.77 |

| Chemical | log $K_{OW}$ | log $k_2$ [$h^{-1}$] | log $BCF_{L5\%}$ | half-life [h] |
|---|---|---|---|---|
| 2,4,5-Trichloroaniline | 3.45 | -0.80 | 1.89 | 4.33 |
| 3-Chloro benzene methanol | 1.94 | -1.05 | 1.30 | 7.70 |
| 2,3,5,6-Tetra chlorophenol | 3.88 | -1.15 | 1.86 | 9.73 |
| S-((5-Methoxy-2-oxo-1,3,4- thiadiazol-3(2H)-yl)methyl) *O,O*- dimethyl ester phosphoro dithioic acid | 2.20 | -0.27 | 0.49 | 1.28 |
| Benzenamine, 2,6-dinitro-*N,N*- dipropyl-4-(tri fluoromethyl)- | 5.34 | -1.35 | 3.30 | 15.55 |
| Benzenamine, 2,6-dinitro-*N,N*- dipropyl-4-(tri fluoromethyl)- | 5.34 | -1.72 | 3.14 | 36.16 |
| Benzenamine, 2,6-dinitro-*N,N*- dipropyl-4-(tri fluoromethyl)- | 5.34 | -2.30 | 3.18 | 138.63 |
| Benzenamine, 2,6-dinitro-*N,N*- dipropyl-4-(tri fluoromethyl)- | 5.34 | -2.02 | 3.11 | 72.33 |
| Benzenamine, 2,6-dinitro-*N,N*- dipropyl-4-(tri fluoromethyl)- | 5.34 | -2.15 | 3.50 | 97.86 |
| Benzenamine, 2,6-dinitro-*N,N*- dipropyl-4-(tri fluoromethyl)- | 5.34 | -2.04 | 3.44 | 75.62 |
| Benzenamine, 2,6-dinitro-*N,N*- dipropyl-4-(tri fluoromethyl)- | 5.34 | -2.02 | 3.53 | 72.33 |
| 2,3,7,8-Tetrachlorodibenzo[b,e][1,4] dioxin | 6.80 | -3.23 | 4.41 | 1188.25 |
| 2,3,7,8-Tetrachlorodibenzo [b,e][1,4]dioxin | 6.80 | -3.30 | 4.58 | 1386.29 |
| 2,3,7,8-Tetrachlorodibenzo [b,e][1,4]dioxin | 6.80 | -3.46 | 4.91 | 2004.28 |
| 2,3,7,8-Tetrachlorodibenzo [b,e][1,4]dioxin | 6.80 | -3.27 | 4.41 | 1279.66 |
| 2,3,7,8-Tetrachlorodibenzo [b,e][1,4]dioxin | 6.80 | -3.30 | 4.62 | 1386.29 |
| 2,3,7,8-Tetrachlorodibenzo [b,e][1,4]dioxin | 6.80 | -2.69 | 3.94 | 339.50 |
| 2,3,7,8-Tetrachlorodibenzo [b,e][1,4]dioxin | 6.80 | -2.71 | 4.61 | 353.95 |
| 1,3-Benzene dicarbonitrile, 2,4,5,6-tetrachloro- | 3.05 | -1.40 | 1.27 | 17.33 |
| Decachloro biphenyl | 8.18 | -3.68 | 4.13 | 3327.11 |
| Ethyl *p*-nitro phenyl phenyl phosphono thioate | 4.78 | -1.70 | 3.07 | 34.66 |
| Ethyl *p*-nitro phenyl phenyl phosphono thioate | 4.78 | -0.74 | 1.83 | 3.85 |
| Bromophos | 5.21 | -1.86 | 3.35 | 50.41 |
| Dimethylphosphoric acid 3- methyl-4-nitrophenyl | 1.69 | -0.52 | 0.49 | 2.31 |
| Mirex | 6.89 | -3.72 | 4.20 | 3616.42 |
| *O*-(2-Chloro-4-nitrophenyl) *O,O*- dimethyl ester phosphorothioic acid | 3.72 | -0.98 | 1.65 | 6.55 |
| alpha- [(Dimethoxyphosphinothioyl) thio]benzene acetic acid, ethyl ester | 3.69 | -0.28 | 1.46 | 1.33 |
| *O*-(4-Cyano phenyl) *O,O*-dimethyl ester, phosphorothioic acid | 2.71 | -0.76 | 1.32 | 3.96 |
| Octahydro-1,3,5,7-tetra nitro- 1,3,5,7-tetrazocine (HMX) | 0.19 | -0.91 | 0.02 | 5.64 |
| Phosphorothioic acid, *O,O*- diethyl *O*-(3,5,6-trichloro-2- pyridinyl) ester | 4.96 | -1.67 | 2.52 | 32.62 |
| Phosphorothioic acid, *O,O*- diethyl *O*-(3,5,6-trichloro-2- pyridinyl) ester | 4.96 | -1.78 | 2.69 | 41.59 |
| Phosphorothioic acid, *O,O*- diethyl *O*-(3,5,6-trichloro-2- pyridinyl) ester | 4.96 | -1.72 | 2.93 | 36.16 |
| Phosphorothioic acid, *O,O*- diethyl *O*-(3,5,6-trichloro-2- pyridinyl) ester | 4.96 | -1.69 | 2.94 | 33.95 |

| Chemical | log $K_{OW}$ | log $k_2$ [$h^{-1}$] | log $BCF_{L5\%}$ | half-life [h] |
|---|---|---|---|---|
| Phosphorothioic acid, *O*,*O*- diethyl *O*-(3,5,6-trichloro-2- pyridinyl) ester | 4.96 | -1.90 | 3.24 | 55.45 |
| Phosphorothioic acid, *O*,*O*- diethyl *O*-(3,5,6-trichloro-2- pyridinyl) ester | 4.96 | -1.98 | 3.27 | 66.54 |
| Phosphorothioic acid, *O*,*O*- diethyl *O*-(3,5,6-trichloro-2- pyridinyl) ester | 4.96 | -1.29 | 3.03 | 13.52 |
| Phosphorothioic acid, *O*,*O*- diethyl *O*-(3,5,6-trichloro-2- pyridinyl) ester | 4.96 | -1.70 | 2.67 | 34.66 |
| Phosphorothioic acid, *O*,*O*- diethyl *O*-(3,5,6-trichloro-2- pyridinyl) ester | 4.96 | -1.69 | 3.24 | 33.95 |
| 1,2,3,4,5,6,7,8-Octachlorodibenzo-*p*-dioxin | 8.20 | -2.30 | 3.90 | 138.63 |
| *O*,*O*-Dimethyl *O*-[3-methyl-4-(methylsulfinyl)phenyl]ester phosphorothioic acid | 1.93 | -0.62 | 0.02 | 2.89 |
| *O*,*O*-Dimethyl *O*-[3-methyl-4-(methylsulfonyl)phenylester phosphorothioic acid | 2.05 | -0.80 | 0.49 | 4.33 |
| 2-Methoxy-4H-1,3,2-benzodi oxaphosphorin-2-sulfide | 2.67 | -0.27 | 0.86 | 1.28 |
| 2-Propanol, 1,3-dichloro-, phosphate (3:1) | 3.65 | -0.38 | 1.86 | 1.65 |
| 2,4,5-Trichloro-1,1'-biphenyl | 5.60 | -2.58 | 3.67 | 264.06 |
| 2,4',5-Trichloro-1,1'-biphenyl | 5.67 | -2.58 | 3.97 | 264.06 |
| Phosphorothioic acid, *O*-(2,5- dichloro-4-iodophenyl) *O*,*O*- dimethyl ester | 5.51 | -1.82 | 3.38 | 46.21 |
| 1,3,5-Trichloro-2-nitrobenzene | 3.69 | -1.93 | 2.66 | 59.41 |
| Phosphorothioic acid, *O*,*O*- diethyl-*O*-(5-phenyl-3-isoxazolyl)ester | 3.73 | -1.52 | 2.63 | 23.10 |
| 1,2,3,7,8,9-Hexachlorodibenzo- p-dioxin | 7.30 | -2.51 | 3.63 | 224.80 |
| 3-[2,4-Dichloro-5-(1-methyl ethoxy)phenyl]-5-(1,1-dimethyl ethyl)-1,3,4-oxadiazol-2(3H)-one | 4.80 | -1.90 | 3.10 | 55.45 |
| Diethylcarbamothioic acid, S-[(4- chloro phenyl)methyl] ester | 3.40 | -0.72 | 2.49 | 3.65 |
| 1,2,3,4-Tetrachlorodibenzo-*p*- dioxin | 6.60 | -1.30 | 3.41 | 13.86 |
| 2,4-Dichloro-1-(3-methoxy-4- nitrophenoxy) benzene | 4.40 | -2.06 | 3.61 | 79.22 |
| *O*,*O*-Dimethyl-*O*-phenyl phosphoro thioate | 3.00 | -1.19 | 1.65 | 10.80 |
| 2,7-Dichloro dibenzo[b,e] [1,4]dioxin | 5.75 | -1.20 | 2.38 | 11.09 |
| 2,2',4,4',5,5'-Hexachloro-1,1'-biphenyl | 6.92 | -4.08 | 5.45 | 8317.77 |
| 2,2',4,4',5,5'-Hexachloro-1,1'-biphenyl | 6.92 | -4.08 | 5.64 | 8317.77 |
| 2,2',4,4',5,5'-Hexachloro-1,1'-biphenyl | 6.92 | -4.08 | 5.66 | 8317.77 |
| 2,2',5,5'-Tetrachloro-1,1'- biphenyl | 5.84 | -3.18 | 4.72 | 1039.72 |
| 2,2',5,5'-Tetrachloro-1,1'- biphenyl | 5.84 | -3.02 | 4.62 | 723.28 |
| 2,2',5,5'-Tetrachloro-1,1'- biphenyl | 5.84 | -3.06 | 4.63 | 792.17 |
| 2,2',5,5'-Tetrachloro-1,1'- biphenyl | 5.84 | -3.02 | 4.66 | 723.28 |
| 1,2,3,4,6,7,8-Heptachlorodibenzo-p-dioxin | 7.80 | -2.47 | 3.38 | 205.38 |
| 1,2,3,4,5,6,7,8-Octachlorodibenzofuran | 8.20 | -2.15 | 2.60 | 97.86 |
| 1,2,3,4,7,8-Hexachlorodibenzo- p-dioxin | 7.30 | -2.57 | 3.71 | 255.93 |
| 1,2,4-Trichlorodibenzo[b,e][1,4]dioxin | 6.35 | -1.42 | 2.64 | 18.28 |

| Chemical | log $K_{OW}$ | log $k_2$ [$h^{-1}$] | log $BCF_{L5\%}$ | half-life [h] |
|---|---|---|---|---|
| 1,2,3,7,8-Pentachlorodibenzo-p- dioxin | 6.64 | -2.90 | 3.97 | 554.52 |
| Bis(1-methyl ethyl)ester, 1,3- dithiolan-2-ylidene propanedioic acid | 2.88 | -0.49 | 1.59 | 2.17 |
| Bis(1-methyl ethyl)ester, 1,3- dithiolan-2-ylidene propanedioic acid | 2.88 | -0.49 | 1.65 | 2.17 |
| Bis(1-methyl ethyl)ester, 1,3- dithiolan-2-ylidene propanedioic acid | 2.88 | -0.72 | 1.45 | 3.65 |
| 2,3,7,8-Tetrachlorodibenzo furan | 6.53 | -1.92 | 2.89 | 57.36 |
| Cyano(3-phenoxyphenyl)methyl ester, 4-chloro-alpha-(1- methylethyl) benzeneacetic acid | 6.20 | -2.04 | 2.64 | 75.62 |
| [1R-[1 alpha(S*),3 alpha]]Cyano(3-phenoxyphenyl)methyl ester 3- (2,2-dibromo ethenyl)-2,2- dimethyl cyclopropane carboxylic acid | 6.20 | -1.86 | 2.30 | 50.41 |
| *O*-(2,6-Dichloro-4-methylphenyl) *O,O*-dimethyl ester, Phosphorothioic acid | 4.56 | -1.40 | 2.36 | 17.33 |
| 2,3,4,7,8-Pentachloro dibenzofuran | 6.64 | -2.74 | 3.84 | 378.08 |
| 1,2,3,6,7,8-Hexachloro dibenzofuran | 7.30 | -2.49 | 3.65 | 213.28 |
| 1,2,3,6,7,8-Hexachloro dibenzo- p-dioxin | 7.30 | -2.68 | 3.64 | 332.71 |
| 2,4,6-tri bromobiphenyl | 6.03 | -2.20 | 3.76 | 110.90 |
| 2,2',5,5'-tetra bromobiphenyl | 6.50 | -3.39 | 4.86 | 1697.50 |
| 2,2',4,4',6,6'-hexa bromobiphenyl | 7.20 | -3.53 | 4.55 | 2343.03 |
| 2,3,4,6,7,8-Hexachloro dibenzofuran | 7.30 | -2.38 | 3.69 | 166.36 |
| *cis*-Permethrin | 7.43 | -1.73 | 2.45 | 36.97 |
| *N*-[3-(1-Methyl ethoxy)phenyl]-2- (trifluoro methyl) benzamide | 3.70 | -0.92 | 1.32 | 5.78 |
| [1 alpha(S*), 3 alpha]-(+-)-3- (2,2-Dichloro ethenyl)-2,2- dimethylcyclo propane carboxylic acid cyano (3- phenoxyphenyl)methyl ester | 6.38 | -2.04 | 2.24 | 75.62 |
| 1,2,3,4,6,7,8-Heptachloro dibenzofuran | 7.40 | -2.27 | 3.16 | 127.97 |
| Phenol, 4-nonyl-, branched, | 5.92 | -1.15 | 2.58 | 9.90 |

**Table S11** Freshwater PNECs (derived from measured single species (*D. magna*) chronic $EC_{10}$s and an assessment factor of 100, using equation: PNEC = $EC_{10}$/assessment factor), environmental concentrations (EC) and risk quotients (RQ) for tested NSO-PAHs.

| | PNEC [ng $L^{-1}$] / [pmol $L^{-1}$] | EC [µg $L^{-1}$] | RQ (EC/PNEC) |
|---|---|---|---|
| BCRB | 150 / 692 | 600 [A] | 4000 |
| BNF | 9.1 / 42 | 190 [B] | 21111 |
| BNT | 3.3 / 14 | 10 [C] | 3333 |
| DNF | 1.4 / 5.2 | 0.8 [B] | 800 |

Environmental concentration (EC) data are obtained from [A] CRB, [B] DBF, and [C] DBT by choosing the highest concentration that remains below the solubility limit of the respective NSO-PAH, as described in Reference [24].

## References


(1) Zhang, G.; Yang, C.; Serhan, M.; Koivu, G.; Yang, Z.; Hollebone, B.; Lambert, P.; Brown, C. E. Characterization of Nitrogen-Containing Polycyclic Aromatic Heterocycles in Crude Oils and Refined Petroleum Products. In *Advances in Marine Biology*; Elsevier Ltd., 2018; Vol. 81, pp 59–96. https://doi.org/10.1016/bs.amb.2018.09.006.

(2) Schwarz, M. A.; Behnke, A.; Brandt, M.; Eisenträger, A.; Hassauer, M.; Kalberlah, F.; Seidel, A. Semipolar Polycyclic Aromatic Compounds: Identification of 15 Priority Substances and the Need for Regulatory Steps under REACH Regulation. *Integr. Environ. Assess. Manag.* **2014**, *10* (3), 415–428. https://doi.org/10.1002/ieam.1526.

(3) OECD. Guidelines for the Testing of Chemicals 305: Bioaccumulation in Fish: Aqueous and Dietary Exposure. **2012**.

(4) Bleeker, E. A. J.; Verbruggen, E. M. J. Bioaccumulation of Polycyclic Aromatic Hydrocarbons in Aquatic Organisms. *Natl. Inst. Public Heal. Environ. (RIVM Rep. 601779002/2009)* **2009**, 1–51. https://doi.org/10.1016/S0304-4203(96)00042-4.

(5) Granier, L. K.; Lafrance, P.; Campbell, P. G. C. An Experimental Design to Probe the Interactions of Dissolved Organic Matter and Xenobiotics : Bioavailability of Pyrene and 2,2′,5,5′-Tetrachlorobiphenyl to Daphnia Magna. *Chemosphere* **1999**, *38* (2), 335–350. https://doi.org/10.1016/S0045-6535(98)00187-8.

(6) Fiedler, Heidelore; Mertens, Christina; Morgenstern, Matthias; Scheidt, Markus; Hutzinger, O. Stoffverhalten von Gaswerkspezifischen Polycyclischen Aromatischen Kohlenwasserstoffen. *Landesanstalt für Umweltschutz Baden-württemb.* **1997**.

(7) de Voogt, P.; van Hattum, B.; Leonards, P.; Klamer, J. C.; Govers, H. Bioconcentration of Polycyclic Heteroaromatic Hydrocarbons in the Guppy (Poecilia Reticula). *Aquat. Toxicol.* **1991**, *20*, 169–194. https://doi.org/10.1016/0166-445X(91)90015-2.

(8) Southworth, G. R.; Beauchamp, J. J.; Schmieder, P. K. Bioaccumulation of Carbazoles: A Potential Effluent from Synthetic Fuels. *Bull. Environ. Contam. Toxicol.* **1979**, *23*, 73–78. https://doi.org/10.1007/BF01769919.

(9) Eastmond, D. A.; Booth, G. M.; Lee, M. L. Toxicity, Accumulation, and Elimination of Polycyclic Aromatic Sulfur Heterocycles in Daphnia Magna. *Arch. Environ. Contam. Toxicol.* **1984**, *13*, 105–111. https://doi.org/10.1007/BF01055652.

(10) Hawker, D. W.; Connell, D. W. Bioconcentration of Lipophilic Compounds by Some Aquatic Organisms. *Ecotoxicol. Environ. Saf.* **1986**, *11* (2), 184–197. https://doi.org/10.1016/0147-6513(86)90063-1.

(11) Atlanta (GA): Agency for Toxic Substances and Disease Registry (US). *Toxicological Profile for Hexachlorobenzene Available from : Https://Www.Ncbi.Nlm.Nih.Gov/Books/NBK592601/*; 2015.

(12) National Library of Medicine (NLM). *Hazardous Substances Data Bank (HSDB)*; Bethesda, MD, 2011. http://toxnet.nlm.nih.gov.

(13) Geyer, H. J.; Scheunert, I.; Brüggemann, R.; Steinberg, C.; Korte, F.; Kettrup, A. QSAR for Organic Chemical Bioconcentration in Daphnia, Algae, and Mussels. *Sci. Total Environ.* **1991**, *109–110*, 387–394. https://doi.org/10.1016/0048-9697(91)90193-I.

(14) Arnot, J. A.; Gobas, F. A. P. C. A Generic QSAR for Assessing the Bioaccumulation Potential of Organic Chemicals in Aquatic Food Webs. *QSAR Comb. Sci.* **2003**, *22* (3), 337–345. https://doi.org/10.1002/qsar.200390023.

(15) Ellgehausen, H.; Guth, J. A.; Esser, H. O. Factors Determining the Bioaccumulation Potential of Pesticides in the Individual Compartments of Aquatic Food Chains. *Ecotoxicol. Environ. Saf.* **1980**, *4* (2), 134–157. https://doi.org/10.1016/0147-6513(80)90015-9.

(16) Newsted, J. L.; Giesy, J. P. Predictive Models for Photoinduced Acute Toxicity of Polycyclic Aromatic Hydrocarbons to Daphnia Magna, Strauss (Cladocera, Crustacea). *Environ. Toxicol. Chem.* **1987**, *6* (6), 445–461. https://doi.org/10.1002/etc.5620060605.

(17) Schlechtriem, C.; Kampe, S.; Bruckert, H. J.; Bischof, I.; Ebersbach, I.; Kosfeld, V.; Kotthoff, M.; Schäfers, C.; L'Haridon, J. Bioconcentration Studies with the Freshwater Amphipod Hyalella Azteca: Are the Results Predictive of Bioconcentration in Fish? *Environ. Sci. Pollut. Res.* **2019**, *26* (2), 1628–1641. https://doi.org/10.1007/s11356-018-3677-4.

(18) Schlechtriem, C.; Kühr, S.; Müller, C. Development of a Bioaccumulation Test Using Hyalella Azteca - Final Report. *Umweltbundesamt* **2021**, 1–135.

(19) OECD. Multi-Laboratory Ring Trial to Support Development of OECD Test Guideline 321 on Hyalella Azteca Bioconcentration Test (HYBIT). *OECD Ser. Test. Assessment, No. 398, OECD Publising, Paris.* **2024**.

(20) Kosfeld, V.; Fu, Q.; Ebersbach, I.; Esser, D.; Schauerte, A.; Bischof, I.; Hollender, J.; Schlechtriem, C. Comparison of Alternative Methods for Bioaccumulation Assessment: Scope and Limitations of In Vitro Depletion Assays with Rainbow Trout and Bioconcentration Tests in the Freshwater Amphipod Hyalella Azteca. *Environ. Toxicol. Chem.* **2020**, *39* (9), 1813–1825. https://doi.org/10.1002/etc.4791.

(21) Lee, J. H.; Landrum, P. F.; Koh, C. H. Toxicokinetics and Time-Dependent PAH Toxicity in the Amphipod Hyalella Azteca. *Environ. Sci. Technol.* **2002**, *36* (14), 3124–3130. https://doi.org/10.1021/es011201l.

(22) Nuutinen, S.; Landrum, P. F.; Schuler, L. J.; Kukkonen, J. V. K.; Lydy, M. J. Toxicokinetics of Organic Contaminants in Hyalella Azteca. *Arch. Environ. Contam. Toxicol.* **2003**, *44* (4), 467–475. https://doi.org/10.1007/s00244-002-2127-x.

(23) Brooke, D.; Crookes, M. Depuration Rate Constant: Growth Correction and Use as an Indicator of Bioaccumulation Potential. *Environ. Agency, UK* **2012**, 1–132.

(24) Çelik, G.; Stolte, S.; Markiewicz, M. NSO-Heterocyclic PAHs - Controlled Exposure Study Reveals High Acute

Aquatic Toxicity. *J. Hazard. Mater.* **2023**, 132428. https://doi.org/doi.org/10.1016/j.jhazmat.2023.132428.
